# CTDP1 governs a Pol II phosphorylation intermediate and coordinates Mediator recruitment, transcription efficiency, and splicing

**DOI:** 10.1093/nar/gkag772

**Published:** 2026-08-06

**Authors:** Haonan Zheng, Dexun Ji, Yuanhan Sui, Qiqin Xu, En-Min Li, Xiong Ji

**Affiliations:** State Key Laboratory of Gene Function and Modulation Research, Beijing Advanced Center of RNA Biology (BEACON), School of Life Sciences, Peking-Tsinghua Center for Life Sciences, Peking University, Beijing 100871, China; Department of Plastic Surgery and Burn Center, The Second Affiliated Hospital of Shantou University Medical College, Shantou, Guangdong 515000, China; State Key Laboratory of Gene Function and Modulation Research, Beijing Advanced Center of RNA Biology (BEACON), School of Life Sciences, Peking-Tsinghua Center for Life Sciences, Peking University, Beijing 100871, China; State Key Laboratory of Gene Function and Modulation Research, Beijing Advanced Center of RNA Biology (BEACON), School of Life Sciences, Peking-Tsinghua Center for Life Sciences, Peking University, Beijing 100871, China; State Key Laboratory of Gene Function and Modulation Research, Beijing Advanced Center of RNA Biology (BEACON), School of Life Sciences, Peking-Tsinghua Center for Life Sciences, Peking University, Beijing 100871, China; Department of Plastic Surgery and Burn Center, The Second Affiliated Hospital of Shantou University Medical College, Shantou, Guangdong 515000, China; State Key Laboratory of Gene Function and Modulation Research, Beijing Advanced Center of RNA Biology (BEACON), School of Life Sciences, Peking-Tsinghua Center for Life Sciences, Peking University, Beijing 100871, China

## Abstract

RNA Pol II exists in hypo- (II-A) and hyper-phosphorylated (II-O) states on chromatin, which are linked to transcription initiation and elongation, respectively. Here, we identify a phosphorylation intermediate of Pol II (designated II-I) that is predominantly localized in the nucleoplasm and is enriched for phosphorylated Tyr1, Ser5, and Ser7 within the C-terminal domain (CTD). We demonstrate that the abundance of this phosphorylation intermediate is rapidly modulated by the phosphatase activity of CTDP1. Mechanistically, CTDP1-mediated regulation is critical for the recruitment of the Mediator complex to chromatin and for modulating Pol II transcriptional efficiency. Moreover, CTDP1’s regulatory function extends to the dephosphorylation of splicing factors. Consequently, its depletion causes widespread intron retention and aberrant splicing of cassette exons. These findings position CTDP1 as a key regulator of Pol II phosphorylation intermediate and splicing regulation, with potential implications for cell cycle control.

## Graphical Abstract

**Graphical Abstract:**
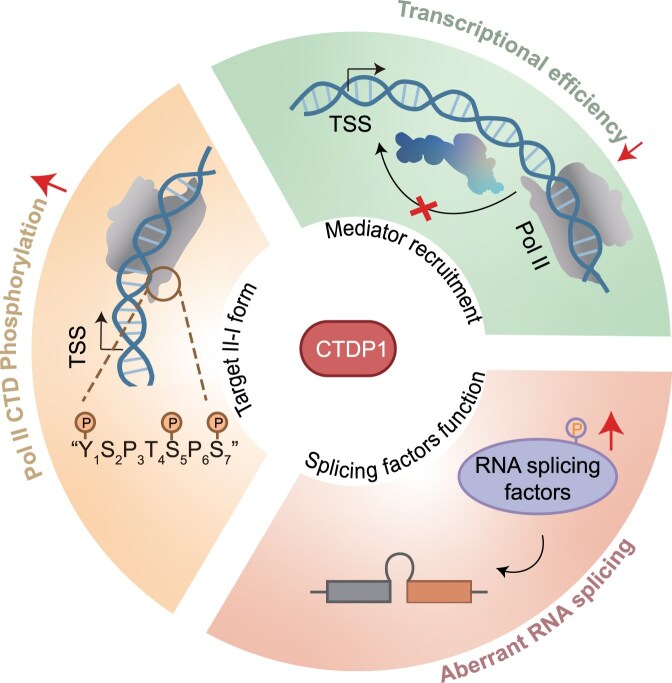

## Introduction

RNA Pol II is widely recognized as a central regulatory machinery governing transcriptional progression [1–5]. In mammalian cells, RNA polymerase II enters a promoter-proximal paused state. As part of the transition from the unphosphorylated or hypophosphorylated Pol II-A form to the hyperphosphorylated Pol II-O at chromatin, the C-terminal domain (CTD) undergoes phosphorylation at tyrosine 1 (Tyr1), serine 5 (Ser5), and serine 7 (Ser7) [6–8]. Pol II pausing after initiation enables co-transcriptional capping, a process that appears to be partially dependent on the CTD [9–11]. This implies the existence of CTD-independent regulation, potentially mediated by the phosphorylation of associated transcription and RNA-metabolic regulators. The subsequent transition to productive elongation is marked by increased phosphorylation of Pol II-O at serine 2 (Ser2) and threonine 4 (Thr4) [12–14], modifications that are associated with diverse co-transcriptional and post-transcriptional RNA processing events, including splicing, 3′ end cleavage, and polyadenylation. Consequently, the prevailing transcriptional regulation framework, which focuses predominantly on classical Pol II CTD phospho-sites, requires expansion beyond chromatin. A comprehensive understanding demands a systematic investigation of the full substrate repertoire of kinases and phosphatases that act beyond the canonical II-A and II-O states.

While previous studies have extensively characterized kinases responsible for Pol II phosphorylation, there is growing interest in the phosphatases that counterbalance these modifications [15]. Several phosphatases have been implicated in Pol II regulation. For instance, the PNUTS-PP1 complex promotes pause release and termination through dephosphorylation of transcription-associated factors such as MEPCE, thereby enhancing elongation efficiency [16–19]. The Integrator-associated PP2A module (INTAC) dephosphorylates the Pol II CTD and SPT5 to reinforce promoter-proximal pausing and restrict productive elongation [20–25]. Our recent work demonstrated that the phosphatase CTDP1 interacts with the Pol II subunit RPB7, stabilizing the polymerase complex, facilitating its dephosphorylation, and enabling transcription reinitiation [26]. In addition, recent reports indicate that RUVBL2 and PP2A are key regulators of Pol II [27, 28]. Here, we employ CTDP1 as a model system to delineate its substrate specificity and elucidate its potential biological functions.

We demonstrate that CTDP1 regulates a specific phosphorylation intermediate of Pol II. We show that this CTDP1-dependent phospho-state is predominantly localized in the nucleoplasm rather than chromatin-bound. Furthermore, we provide evidence that CTDP1-mediated regulation is critical for Mediator complex chromatin occupancy and transcriptional efficiency. Through integrated interaction proteomics and phosphoproteomics, we identified multiple splicing factors as CTDP1 targets and established that CTDP1 depletion leads to widespread alternative splicing defects. Collectively, our findings support a model in which CTDP1 modulates the Pol II phosphorylation intermediate and coordinates transcription with RNA processing, thereby influencing cell cycle progression.

## Materials and methods

### Cell culture

CTDP1 degron cells were V6.5 mouse embryonic stem cells maintained in Dulbecco’s modified Eagle’s medium (DMEM) supplemented with 15% fetal bovine serum (FBS), nonessential amino acids, nucleosides, penicillin/streptomycin, 0.1 mM β-mercaptoethanol, 1 mM sodium pyruvate, 1 μM PD0325901, 3 μM CHIR99021, and 1000 U/ml mouse leukemia inhibitory factor. The cells were cultured on 0.2% gelatin-coated plates at 37°C in a humidified atmosphere with 5% CO_2_. *Drosophila* S2 cells for RNA-seq spike-in were grown in Schneider’s *Drosophila* Medium containing 10% FBS and incubated at 28°C. HEK293T cells used for co–immunoprecipitation were maintained in DMEM supplemented with 10% FBS and penicillin/streptomycin. HEK293F cells were grown in SMM 293–TII Expression Medium (Sinobiological, M293TII) under shaking conditions at 120 rpm. All cells were confirmed to be free of mycoplasma contamination before experimental use.

### Cell lines generation

To integrate expression cassettes into the *Tigre* locus, we co-transfected CTDP1 degron cells [26] or GFP-RPB1 cells [29] with the following constructs: a Cas9-GFP plasmid expressing a sgRNA targeting the *Tigre* locus, and donor vectors carrying either wild-type (WT) or MT CTDP1 fused to mCherry-Flag under a TRE promoter, together with a hygromycin resistance (HygR) gene driven by a PGK promoter. Transfection was performed using FuGENE HD. Post-transfection, cells were selected with hygromycin B, and mCherry-positive populations were isolated. Successful knock-in clones were subsequently verified by western blot to confirm Flag-tagged CTDP1 expression.

### ChIP-seq and ChIP-qPCR

ChIP-seq was conducted following a previously described methodology [26], with two biological replicates included per sample. In brief, 20 million cells supplemented with 10% HEK293T cells were harvested by trypsinization and crosslinked with 1% formaldehyde for 10 min at room temperature. The crosslinking reaction was quenched by adding glycine to a final concentration of 0.125 M and incubating for 5 min. Cells were lysed on ice for 5 min using 0.5 ml of ice-cold NP-40 lysis buffer (10 mM Tris–HCl, pH 7.5, 150 mM NaCl, 0.05% IGEPAL® CA-630), then layered over a 1.25 ml sucrose cushion (24% sucrose in NP-40 lysis buffer) and centrifuged at 13 000 × *g* for 10 min at 4°C. The supernatant was discarded, and the nuclear pellet was washed once with 1 ml phosphate buffered saline (PBS)/1 mM ethylenediaminetetraacetic acid (EDTA). Nuclei were resuspended in glycerol buffer (20 mM Tris–HCl, pH 8.0, 75 mM NaCl, 0.5 mM EDTA, 0.85 mM DTT, 50% glycerol), followed by the addition of an equal volume of nuclear lysis buffer (10 mM HEPES, pH 7.6, 1 mM DTT, 7.5 mM MgCl_2_, 0.2 mM EDTA, 0.3 M NaCl, 1 M urea, 1% IGEPAL® CA-630) and incubation on ice for 2 min. The chromatin pellet was collected by centrifugation at 13 000 × *g* for 2 min, washed twice with PBS/1 mM EDTA, and resuspended in 1 ml sonication buffer [20 mM Tris–HCl, pH 8.0, 5 mM CaCl_2_, 150 mM NaCl, 2 mM EDTA, 0.1% sodium dodecyl sulfate (SDS), 1% Triton X-100]. Chromatin was digested with 1000 U of MNase for 15 min at 37°C with shaking at 700 rpm. The reaction was stopped by adding 20 μl of 0.5 M EDTA and 40 μl of 0.5 M ethylene glycol-bis(β-aminoethyl ether)-N,N,N′,N′-tetraacetic acid (EGTA). The sample was aliquoted into 300 μl portions and sonicated using a Qsonica system (70% amplitude, 30 s on/60 s off, 20 cycles). After two rounds of centrifugation at 13 000 × *g* for 10 min at 4°C, the supernatant was transferred to a new tube, and 20 μl was reserved as input. For each ChIP reaction, 1 μg antibodies were added. Lysates were incubated overnight at 4°C with rotation, centrifuged, and transferred to tubes containing 30 μl of pre-washed Protein G magnetic beads for a 3-h incubation at 4°C with rotation. Beads were sequentially washed with sonication buffer, high-salt wash buffer (20 mM Tris–HCl, pH 8.0, 500 mM NaCl, 2 mM EDTA, 0.1% SDS, 1% Triton X-100), LiCl wash buffer (10 mM Tris–HCl, pH 8.0, 250 mM LiCl, 1 mM EDTA, 1% NP-40), and TE buffer (1 mM EDTA, 10 mM Tris–HCl, pH 8.0, three times). Bound complexes were eluted twice with 150 μl elution buffer (50 mM Tris–HCl, pH 8.0, 10 mM EDTA, 1% SDS) at 65°C with shaking at 900 rpm. The eluted ChIP DNA, along with input samples, was treated with 5 μl Proteinase K overnight at 65°C, followed by enzyme inactivation at 80°C for 20 min. DNA was purified using DNA extraction reagent, and libraries were constructed with the NEBNext Ultra II DNA Library Prep Kit according to the manufacturer’s instructions. Sequencing was performed by Novogene on a NovaSeq platform (PE150). In DRB release/pSer2 RPB1 ChIP-seq experiments, cells were pretreated with 100 μM DRB for 3.5 h, washed twice with PBS to remove DRB, and incubated in fresh medium at 37°C for 0, 10, 20, or 40 min before immediate processing for ChIP-seq. For CTDP1 depletion studies, 500 μM auxin was added after 1.5 h of DRB treatment and maintained in the fresh medium thereafter. For ChIP-quantitative polymerase chain reaction (qPCR) analysis, DNA samples isolated prior to library construction were amplified using SYBR qPCR Master Mix on a Bio-Rad CFX Connect™ Real-Time PCR Detection System.

### Subcellular fractionation

To assess protein level changes in distinct compartments, we performed subcellular fractionation to isolate soluble and chromatin-bound fractions following a standard ChIP-seq guideline [26]. In brief, ~10 million cells were gently detached from culture plates, transferred to 1.5 ml tubes, and pelleted by centrifugation (300 × *g*, 4°C, 3 min). Cell pellets were washed twice with 1 ml of PBS containing 1 mM EDTA, then sequentially resuspended in 0.5 ml glycerol buffer (20 mM Tris–HCl, pH 8.0, 75 mM NaCl, 0.5 mM EDTA, 0.85 mM DTT, 50% glycerol) and 0.5 ml nuclei lysis buffer (10 mM HEPES, pH 7.6, 1 mM DTT, 7.5 mM MgCl_2_, 0.2 mM EDTA, 0.3 M NaCl, 1 M urea, 1% IGEPAL® CA-630), followed by incubation on ice for 2 min. After centrifugation at 13 000 × *g* for 2 min at 4°C, the supernatant was collected as the soluble nuclear fraction (Sup). The resulting chromatin pellet was washed with 1 ml PBS/1 mM EDTA and subjected to sonication in 0.5 ml of the same buffer using a Qsonica Q800R3 system (30% amplitude, 30 s on/30 s off, 4 cycles). The homogenized suspension was designated as the chromatin-enriched fraction (Chr).

### Western blot analysis

Western blotting was performed with a minimum of two biological replicates per condition. Protein samples were denatured by heating at 100°C for 10 min in 2 × SDS loading buffer (125 mM Tris–HCl, pH 6.8, 4% SDS, 20% glycerol, 10% β-mercaptoethanol, 0.01% bromophenol blue) and separated by SDS–polyacrylamide gel electrophoresis (PAGE). To resolve the Pol II II-I form, the 6% separating gel was used, and electrophoresis was carried out initially at 80 V for 30 min, followed by 120 V until the 100 kDa protein standard reached the bottom of the gel. Proteins were subsequently transferred to a PVDF membrane electrophoretically at 200 mA for 4 h in an ice bath. The membrane was blocked with 5% (w/v) skim milk in TBST [Tris-Buffered Saline (TBS) containing 0.1% Tween-20] for 30 min at room temperature, then incubated with primary antibodies against target proteins overnight at 4°C. After three washes with TBST, the membrane was probed with corresponding HRP-conjugated secondary antibody diluted 1:5000 in TBST with 5% skim milk for 1 h at room temperature, followed by another three TBST washes. Signal detection was performed using an Enhanced/Super ECL kit, and images were acquired with an Amersham Imager 600 system (GE Healthcare Life Sciences).

### Co–immunoprecipitation assays

Approximately 10 million cells per replicate were gently harvested by scraping and lysed in 1 ml of Western/IP lysis buffer (Beyotime, P0013) on ice for 15 min. Lysates were centrifuged at 12 000 × *g* for 10 min at 4°C, and the supernatant was collected. An aliquot of 50 μl was reserved as input. The remaining lysate was incubated with anti-FLAG magnetic beads or Strep-Tactin beads for 4 h at 4°C with rotation. All beads were washed three times for 5 min each on a magnetic stand using lysis buffer. Bound proteins were eluted in 2 × SDS loading buffer by heating at 100°C for 10 min. For RPB1-ChIP WB, lysates were prepared following the ChIP-seq protocol described earlier, then the Protein G beads were washed and eluted following the Co–IP procedure described earlier. For *in vitro* calf intestinal alkaline phosphatase (CIP) phosphatase treatment, after extensive washing of the Strep-Tactin beads, the supernatant was removed, and the beads were resuspended in a dephosphorylation reaction buffer [100 mM NaCl, 50 mM Tris–HCl (pH 7.9 at 25°C), 10 mM MgCl_2_, and 1 mM DTT], supplemented with protease inhibitors. For the treatment group, 20 units of CIP were added to the reaction, while the control group received no CIP (all other conditions were identical). The reactions were incubated at 37°C with shaking at 1000 rpm for 1 h. Following incubation, the beads were washed three times with IP wash buffer to remove the enzyme and reaction mixture. Finally, the beads were boiled in 2 × SDS loading buffer at 100°C for 10 min to elute the proteins, which were then subjected to western blot analysis.

### Flow cytometry

GFP-RPB1/Teton MT-CTDP1-FKBP cells were prepared from three treatment conditions: untreated control, DOX-only (13 h), and DOX (12 h) followed by dTAG–13 (1 h). Single–cell suspensions were obtained using 0.25% Trypsin–EDTA. For extraction, cells were treated with CSK buffer (25 mM HEPES, pH 7.4, 50 mM NaCl, 1 mM EDTA, 3 mM MgCl_2_, 300 mM sucrose, 0.5% Triton X-100, protease inhibitor) on ice for 10 min to obtain the insoluble fraction. All suspensions—with or without extraction—were filtered through a 70 µm nylon mesh to remove aggregates and debris. Sample acquisition was performed on a BD LSRFortessa flow cytometer operated with FACSDiva™ Software (v8.0). Data were analyzed using FlowJo (v10.8.1) and GraphPad Prism (v9.5.0).

### Mass spectrometry analysis

For Pol II ChIP-MS, cells were processed following the standard ChIP-seq protocol, including collection, lysis, enrichment, and bead washing. Then co-precipitated complexes were subsequently eluted from the beads using 2 × SDS loading buffer at 100°C for 10 min. For CTDP1 IP-MS, the co–immunoprecipitation protocol was applied. All samples were prepared in three biological replicates. Following SDS–PAGE and Coomassie blue staining, gel regions containing enriched proteins were excised and subjected to data–dependent LC–MS/MS analysis on a Thermo Orbitrap Exploris 480 mass spectrometer. Raw data were processed using Proteome Discoverer software (version 2.2) for label-free quantification. For crosslinking mass spectrometry (XL-MS) analysis, MT CTDP1 fused to a Strep-Twin tag was overexpressed in HEK293F cells. Following lysis, the tagged protein was affinity-purified using Streptactin beads, eluted with desthiobiotin, and subjected to size-exclusion chromatography. Fractions corresponding to CTDP1-Pol II complex were collected for subsequent *in vitro* crosslinking with BS3. The crosslinking reaction was quenched with Tris buffer prior to MS sample preparation. For phosphoproteomic sample preparation, the procedure followed the protocol published previously [30].

### Structure alignment

The structural model of the RPB7-50 × GS-CTDP1 fusion protein was predicted using AlphaFold2. Following execution of the run_alphafold.sh script as specified in the GitHub documentation, the highest-confidence predicted structure was selected for further analysis. This model was imported into PyMOL and structurally aligned with the previously reported WT RPB7 structure (PDB: 6xre).

### PRO-seq

PRO-seq was conducted using a 1-biotin run-on system, following an established protocol [31]. Each sample was analyzed in two biological replicates. In brief, 10 million cells were trypsinized and combined with 10% S2 cells as a spike-in control. The cell mixture was permeabilized on ice for 5 min using a buffer containing 10 mM Tris–HCl, pH 7.4, 300 mM sucrose, 10 mM KCl, 5 mM MgCl_2_, 1 mM EGTA, 0.05% Tween-20, 0.1% NP-40, 0.5 mM DTT, 4 U/ml RNase inhibitor, and protease inhibitor cocktail. Nuclei were pelleted, washed twice with the same buffer, and resuspended in 100 μl of storage buffer (10 mM Tris–HCl, pH 8.0, 25% glycerol, 5 mM MgCl_2_, 0.1 mM EDTA, 5 mM DTT) for storage at −80°C. For the run-on reaction, thawed nuclei were mixed with 100 μl of a freshly prepared 2 × reaction mix (10 mM Tris–HCl pH 8.0, 5 mM MgCl_2_, 1 mM DTT, 300 mM KCl, 50 μM biotin-11-CTP, 0.5 μM CTP, 250 μM each of ATP, GTP, and UTP, 80 U RNase inhibitor, 1% Sarkosyl) and incubated at 37°C for 3 min. RNA was immediately extracted with TRIzol LS reagent. After purification, the RNA pellet was dissolved in 200 μl of RNase-free water and fragmented by sonication (Diagenode Bioruptor Plus; 10 cycles of 30 s on/30 s off at high intensity). Biotinylated RNA was enriched by denaturing the fragmented RNA at 65°C for 40 s, followed by incubation with pre-washed streptavidin Dynabeads in 200 μl binding buffer (10 mM Tris–HCl, pH 7.4, 300 mM NaCl, 0.1% Triton X-100) for 20 min at 25°C. The beads were subsequently washed three times with a high-salt buffer (50 mM Tris–HCl, pH 7.4, 2 M NaCl, 0.5% Triton X-100) and once with a low-salt buffer (5 mM Tris–HCl, pH 7.4, 0.1% Triton X-100). Bound RNA was eluted by vortexing the beads twice with 300 μl of TRIzol reagent. The final RNA pellet was dissolved in 8 μl of RNase-free water, and sequencing libraries were constructed using the VAHTS Universal V8 RNA-seq Library Prep Kit according to the manufacturer’s instructions.

### Protein expression and purification

The CTDP1 fusion gene was cloned into an expression vector. The plasmid was transiently transfected into suspension HEK293F cells using polyethylenimine and cultured for ~60 h. Cells were harvested by centrifugation and washed with TBS. Cell pellets were resuspended and lysed in ice-cold Lysis Buffer (50 mM HEPES, pH 7.5, 150 mM NaCl, 5 mM MgCl_2_, 10% glycerol, 1 mM DTT, 1% NP-40, protease inhibitors, and 5 mM ATP) for 1 h at 4°C. The lysate was clarified by centrifugation. The supernatant was incubated with Streptactin-conjugated agarose beads for batch binding. The beads were washed with Wash Buffer (50 mM HEPES pH 7.5, 150 mM NaCl, 5 mM MgCl_2_, 10% glycerol, 1 mM DTT, 0.5% NP-40), and the bound protein was eluted using Elution Buffer containing 2.5 mM desthiobiotin. The eluate was concentrated, and the buffer was exchanged to Storage Buffer (50 mM HEPES, pH 7.5, 150 mM NaCl, 5 mM MgCl_2_, 10% glycerol, 1 mM DTT) using a centrifugal concentrator. The purified protein was used for subsequent assays.

### Phosphatase assay

Reaction mixtures (100 µl) containing 50 mM HEPES (pH 7.5), 20 mM MgCl_2_, 10 mM pNPP, and CTDP1 protein as indicated were incubated for 1 h at 37°C. The reactions were quenched by adding 100 µl of 1 M NaOH. Release of p-nitrophenol (pNP) was determined by measuring the absorbance at 410 nm (A_410_).

### *In vitro* dephosphorylation assay using phosphopeptides

To assess the direct phosphatase activity of CTDP1, an *in vitro* dephosphorylation assay was performed using synthetic phosphopeptides. Reaction mixtures containing 50 mM HEPES (pH 7.5), 20 mM MgCl_2_, 2 mM DTT, 50 µM phosphopeptide, and 10 µg of purified WT CTDP1 protein were incubated at 37°C for 2 h. The reactions were terminated by adding Malachite Green reagent (Beyotime), and the release of inorganic phosphate was quantified by measuring the absorbance at 630 nm.

### *In vitro* transcription recycling assay

*In vitro* transcription recycling assay was prepared as described earlier [32]. Two biological replicates were analyzed for each sample. First, linearized DNA templates were prepared by using 5′-biotinylated forward primers to amplify from p300 and pEF1 plasmids (Addgene: #23252 and #72485) with KOD FX enzyme following the manufacturer’s instructions. After being purified using AMPure XP beads, PCR products were immobilized on DynabeadsTM M280 Streptavidin beads in 0.5 × binding buffer (10 mM Tris–HCl, pH 7.4, 300 mM NaCl and 0.1% Triton X-100) for 3 h at room temperature. After being washed in binding buffer twice and Tris–HCl (pH 8.0) twice, immobilized templates were resuspended in RNase-free water. The nuclear extract was prepared from CTDP1-degraded cells. Briefly, 40 million cells were washed and scraped in cold PBS. After centrifugation, cell pellets were suspended in 2 ml Buffer A (10 mM HEPES, pH 7.9, 3 mM MgCl_2_, 10 mM KCl and 0.5 mM DTT) for 10 min on ice, then lysed by ∼10 strokes of Dounce homogenizer (B type pestle). Nuclei were collected by centrifugation for 10 min at 4000 rpm at 4°C and resuspended in 0.8 ml Buffer C (20 mM HEPES, pH 7.9, 25% glycerol, 0.42 M NaCl, 5 mM MgCl_2_, 0.2 mM EDTA, 0.5 mM DTT, and 1 × Protease Inhibitor Cocktail) for 40 min at 4°C The nuclear extract was collected by centrifugation for 20 min at 20 000 x *g* at 4°C and dialyzed against 50 ml Buffer D (20 mM HEPES, pH 7.9, 20% glycerol, 0.1 M KCl, 0.2 mM EDTA, 0.5 mM DTT, 1 × Protease Inhibitor Cocktail) in Tube-O-DIALYZE^TM^ dialysis device for 12 h, then repeated dialysis twice. After centrifugation, the supernatant was frozen on liquid nitrogen and stored at −80°C. After adding exogenous-purified WT or MT CTDP1, the nuclear extract was incubated with template 1 for 30 min at 30°C at 1000 rpm to form PIC in transcription buffer (10 mM HEPES, pH 7.9, 10% glycerol, 0.25 mM DTT, 0.1 mM EDTA, 50 mM KCI, 3 mM MgCl_2_), then 600 μM NTPs were added to starting the transcription for 1 h at 30°C at 1000 rpm. The supernatant (Round 1) was collected. After being washed twice in ice-cold washing buffer (10 mM HEPES, pH 7.9, 50 mM KCl, 0.1 mM EDTA, 0.25 mM DTT 10% glycerol, 0.05% NP-40) and once without NP-40, the beads were then incubated in new transcription buffer for another 30 min at 30°C. The solution was collected and incubated immediately with the template 2 to allow transcription recycling start. After incubating for 1 h at 30°C, solutions containing the template 2-derived RNA products (Round 2) were collected. The RNA products in two collected samples were extracted by using the PureLink™ RNA Mini Kit, and then converted to complementary DNA by using HiScript IV RT SuperMix for qPCR. RT-qPCR was conducted same as ChIP-qPCR described earlier. All the primers used were the same as described earlier [32].

### RNA-seq

To analyze transcriptomic changes upon the expression of MT-CTDP1, Teton MT-CTDP1 cells were treated with 2 μg/ml Dox for a series of time points: 0, 12, 24, 36, and 48 h. Then, cells from one well of a six-well plate were lysed in 0.5 ml of TRIzol reagent with 10% *Drosophila* S2 cells included as a spike-in control for normalization. Each condition included two biological replicates. Total RNA was extracted according to the manufacturer’s protocol and submitted to Annoroad (Beijing) for poly(A) selection, library preparation, and sequencing.

### Cell cycle

Cells (1 × 10^6^) were collected, centrifuged at 400 × *g* for 4 min, and washed with 1 ml PBS. The pellet was fixed by adding 70% ethanol dropwise with gentle vortexing, and incubated at 4 °C for ≥30 min (usually overnight). After fixation, cells were centrifuged again, washed with PBS, and stained in 0.3 ml PBS containing 50 μg/ml PI and 50 μg/ml RNase for 15 min at room temperature. The suspension was filtered through a 200-mesh nylon mesh and analyzed on a BD LSRFortessa flow cytometer. Data analysis was performed using FlowJo (v10.8.1) and GraphPad Prism (v9.5.0).

### Cell proliferation assay

After cell counting, 3000 cells per well were seeded in 96-well plates with three biological replicates. At each designated time point, Teton WT/MT CTDP1 cells in corresponding experimental groups were treated with 2 μg/ml doxycycline (DOX) for exogenous WT/MT CTDP1 induction, while CTDP1 degron cells were treated with 500 μM auxin. Control groups received an equal volume of fresh culture medium. Finally, CCK-8 reagent was added to each well at a 1:10 volume ratio and incubated for 2 h at 37°C. Absorbance at 450 nm was subsequently measured using a BioTek Cytation5 microplate reader. Wells containing culture medium without cells served as blank controls for background subtraction.

### RNA-seq data analysis

The paired-end sequencing RNA-seq reads were first trimmed by Cutadapt (v3.4) [33], then the filtered reads were aligned to mm10 merged with dm6 genome using STAR (v2.7.10a) with default parameters [34]. PCR duplicates, ribosomal RNA (rRNA) reads and incorrectly paired reads were removed for further analysis. Reads that mapped to each gene were counted by featureCounts (v2.0.1) [35] from the GENCODE annotation (M23). Spike-in reads were quantified in parallel and used to normalize gene-level read counts across samples. Genes with read counts >10 in any sample were selected for downstream analysis. For time-course expression pattern analysis, R package Mfuzz (v2.68.0) [36] was applied to perform fuzzy C-means clustering across the time series. Spike-in-normalized RNA-seq counts were standardized (mean = 0, SD = 1) prior to clustering, and the fuzzification parameter m was estimated using the mestimate function. The optimal number of clusters was selected based on the Dmin curve. Membership scores were calculated to assign genes to dominant temporal profiles, and genes with membership >0.6 in specific cluster were retained for further biological interpretation. Gene Ontology (GO) enrichment analysis was performed using the R package clusterProfiler (v4.16.0) [37]. The alternative splicing analysis was performed with rMATS (v4.1.2) [38]. STAR alignments from the RNA-Seq data (BAM format) were taken as inputs to detect and quantify alternative splicing events with GENCODE annotation. The inclusion levels were calculated by junction reads normalized by effective junction length. The differential analysis of alternative splicing events was performed between each subsequent time point and the CTDP1-MT-0 h control with FDR < 0.05 as cutoff. Hierarchical clustering was applied to characterize temporal patterns of alternative splicing events using the Ward.D2 linkage method. Regarding the differential splicing analysis, the values shown in the density plots of Fig. 5F represent IncLevelDifference (also known as ΔPSI or ΔΨ) [39]. IncLevel (Inclusion Level) represents the proportion of the “inclusion isoform” out of the two types of transcripts for a specific splicing event [e.g. the ratio of transcripts containing the target exon for skipped exon (SE) events, or retaining the intron for retained intron (RI) events]. IncLevelDifference represents the difference in IncLevel, calculated by subtracting the inclusion level of the 0 h control from the value at each specific treatment time point (e.g. IncLevelDifference = IncLevel(timepoint) – IncLevel(0 h)), rather than log_2_(fold change). In common, an absolute IncLevelDifference (ΔPSI) of ± 0.1 is a widely accepted threshold for biologically significant shifts in splicing patterns [40]. For statistical evaluation, the Likelihood Ratio Test implemented in the rMATS (v4.1.2) framework were used [38]. The *P*-values were adjusted using the Benjamini–Hochberg procedure to control the false discovery rate (FDR), and a threshold of FDR < 0.05 was applied to identify significant events without a fixed IncLevelDifference cutoff in Fig. 5F. The total number of identified differential splicing events is detailed in Supplementary Table S3. For Fig. 5G and H, alternative splicing events that were consistently detected across all sampled time points were focused.

### MS data analysis

The peptides were mapped using Proteome Discoverer software. The abundance for each protein was calculated as the sum of the abundance of its related peptide groups. The abundances of the proteins were normalized by the library size of each sample. Prior to downstream analysis, proteins exhibiting missing values across all three biological replicates within any experimental condition were removed, ensuring that only reliably quantified proteins were retained. The differentially enriched proteins in different experimental conditions were identified using the R package DEP (v1.30.0) [41] with a threshold of *P* value < .05 and |log_2_FoldChange| > 1. To generate a network of transcription-related protein complexes associated with CTDP1, complexes enriched under the GO cellular component category were selected from the CTDP1 MT/WT IP-MS dataset. The values of log_2_FoldChange (MT/WT) were used as inputs to generate a network using STRING database in Cytoscape (v3.10.3) [42].

### Phosphoproteomics data analysis

Phosphopeptides were identified and quantified using Proteome Discoverer. The phosphopeptide abundances were normalized according to the library size of each sample. Prior to downstream analysis, phosphopeptides with missing values in two or more of the three biological replicates within any experimental condition were removed. Differential enrichment analysis of phosphopeptides was carried out using the same statistical workflow as applied in the protein-level MS analysis.

### ChIP-seq and PRO-seq alignment

Raw sequencing reads, including spike-in controls, were first processed with Cutadapt (v3.4) [33] to remove adapter sequences. For ChIP-seq, the trimmed reads were then aligned to the mm10 and hg19 reference genomes using Bowtie2 (v2.3.5.1) in end-to-end alignment mode [43]. Only uniquely and concordantly mapped reads were retained for downstream analyses. BigWig files were generated with the bamCoverage function from deepTools (v3.5.1) [44], normalized using reads mapped to the hg19 genome. Meta-gene plots and heatmaps plots were plotted in deepTools using BigWig files merged from both replicates. Active genes were defined as those with RNA-seq read counts >3 in both replicates of untreated RPB7 degron cells. For PRO-seq, rRNA reads were discarded, the remaining reads were then mapped to mm10 and dm6 genome. For the quantification of genomic features and subsequent statistical analyses, read counting was performed with featureCounts (v2.0.1) [35]. Differential binding analysis was carried out using DESeq2 (v1.48.2) [45] with spike–in normalized counts; a two–sided Wald test was applied to identify significant changes across multiple time–point comparisons. GO enrichment analysis was performed with the clusterProfiler R package (v4.16.0) [37]. For the meta–gene plots and heatmaps of H3K27ac and H3K4me3, publicly available data from GEO accessions GSM4886936 and GSM4886930 were used as references, respectively.

### DRB/pSer2 ChIP-seq elongation wave and velocity prediction

To predict a valid elongation wave for each transcript across the genome, the transcripts were firstly selected by meeting the two criteria: 1) ChIP-seq signals of the transcripts were up-regulated compared with 0 min. The up-regulated transcripts were identified by DESeq2 package with a threshold of p.adj < 0.05 & log_2_FoldChange > 1.2) The transcript lengths used to predict wave length at 10, 20, and 40 min wave were longer than 30, 50, and 90 kb, respectively, to ensure that the transcripts were long enough for wave prediction. The elongation waves were then predicted using an adapted version of groHMM [46]. The changes are mainly in adapting the program to non-stranded ChIP-seq data, and the parallel computation to save the running time. After obtaining the wave end for each transcript, we selected the valid wave following the two criteria: (i) The elongation velocity identified at each time point should be faster than 100 bp/min and slower than 10 kb/min [47]. (ii) The mean ChIP-seq signal at transcriptional start site (TSS) to wave end should be larger than that at wave end to transcriptional end site (TES). We also performed a *t*-test to the distribution of ChIP-seq signal at the two regions, and required that the adjusted *P* value should be <0.05. In order to screen out duplicated transcripts, we kept only one transcript for the duplicated transcripts with the same wave end, and selected the longest transcript for a gene if duplication still existed after filtering. The mean elongation velocity of a transcript was calculated by fitting a linear regression to the waves mapped at each timepoint. For the transcripts used for the comparison between untreated and degradation conditions, we selected the transcripts with valid predicted waves at least two time points in both the two conditions.

### Mathematical modeling

In general, the model describes the behavior of Pol II on a model gene of 35 kb in length (median length of the active genes that do not overlap within 10 kb of each other) in several continuous processes. The Pol II loads on to the gene from the effective Pol II pool, then elongates along the gene (process 1). As it elongates it would be phosphorylated and dephosphorylated (process 2), and has a probability to dissociate from the chromatin (process 3). The dropped Pol II should be dephosphorylated before returning to the effective Pol II pool to start a new round of transcription, otherwise, it would be degraded (process 4). At short time intervals, we count the relative number of Pol II that initiates and terminates to update the effective Pol II pool size, and then update the relative initiation frequency according to the effective pool size.

Several parameters were visualized in the model to monitor the dynamics of Pol II phosphorylation in recycling. The pSer2 state per Pol II was described by an exponential function according to previous knowledge [15], which accumulated slowly along gene bodies and rapidly when approaching termination sites. Active Pol II signals were calculated as the proportion of Pol II remaining on the gene when an initial amount of Pol II extends to a certain position in the gene body, normalized by the interval between the two Pol II. The pSer2 state signals were the product of pSer2 state per Pol II and the active Pol II signals. The recycling factor was defined as the effective pool size normalized by the initial effective pool size, because it indicates the number of Pol II that was able to recycle. As mentioned earlier, the effective pool size was updated at regular intervals as the current pool size minus the relative number of Pol II that initiates, plus the portion of the dropped Pol II that was dephosphorylated.

Based on the experimental results we knew that three processes should be altered after CTDP1 degradation, that is, the increase of pSer2 signals, the destabilization of RPB1 that is incapable of being dephosphorylated, and the increase of elongation rate. Therefore, we set the pSer2 state per Pol II in process 2 and the elongation velocity in process 1 to increase, and the proportion of Pol II that was dephosphorylated and capable of returning to the effective Pol II pool in process 4 to decrease in the model. And the pSer2 state signals, which were similar to pSer2 ChIP-Seq signals, were reconstructed and compared with the change of real pSer2 ChIP-Seq signal after CTDP1 degradation. We discussed the model in detail in the following sections. Note that the polymerases were assumed to be independent of and not supposed to bump with each other.

In process 1, the moving trajectory of Pol II is described following the mathematical theory of traffic flow [48]. For a queue of Pol II that loads on to the gene at a time ${t_i}$ and moves at a certain location $x$ at a given time $t$, the velocity of the polymerase is described by a function ${k_{el}}(x,t)$. The moving trajectory $x(t,{t_i})$ of any given polymerase can be obtained by solving the ODE.


\begin{eqnarray*}
\frac{{\mathrm{ d}x}}{{\mathrm{ d}t}} = {k_{el}}\left( {x\left( t \right),t} \right),x\left( {{t_i}} \right) = 0.
\end{eqnarray*}

Previous studies have discovered that polymerases accelerate during elongation and decelerate upon termination [47, 49]. So for simplicity, we assigned a segmented quadratic function to ${k_{el}}( {x,t} )$.


\begin{eqnarray*}
{k_{el}}\left( {x,t} \right) = \left\{ {\begin{array}{@{}*{2}{l}@{}} {{\beta _1}{{\left( {x - {\omega _1}} \right)}^2} + \varphi \left( t \right),}&{x \le {\omega _1}}\\ {\varphi \left( t \right),}&{{\omega _1} < x \le {\omega _2}}\\ {{\beta _2}{{\left( {x - {\omega _2}} \right)}^2} + \varphi \left( t \right),}&{x > {\omega _2}} \end{array}} \right..
\end{eqnarray*}

Where $\varphi ( t )$ is the maximum speed the polymerases achieve, and is set increased upon the degradation of CTDP1 according to DRB/pSer2 ChIP-seq experiment. $\beta $ is the acceleration or deceleration speed and is updated according to the increase of $\varphi ( t )$. $\omega $ is the position of polymerases reaching maximum speed or starting to deceleration and is not supposed to change over time.

In process 2, as mentioned earlier for simplicity, we hypothesized an exponential increase of pSer2 state per Pol II which accumulated slowly along gene bodies and rapidly when approaching termination sites. At any given time, the pSer2 state per Pol II at a given position is normalized to that at the start site ($x = 0$), we have


\begin{eqnarray*}
{U_{p{\mathrm{ho}}}}\left( x \right) = {U_{p{\mathrm{ho}}}}\left( 0 \right)\cdot\left( {{e^{\theta \left( {x - \gamma } \right)}} + 1 - {e^{ - \theta \gamma }}} \right).
\end{eqnarray*}

Where $1 - {e^{ - \theta \gamma }}$ is used to control that ${U_{p{\mathrm{ho}}}}( 0 ) \equiv 1$. We knew from our investigation that the pSer2 state per Pol II increases upon the degradation of CTDP1. So the overall increasement of ${U_{p{\mathrm{ho}}}}( x )$ can be described by multiplying a ratio that >1, and the ratio can be combined in to ${U_{p{\mathrm{ho}}}}( 0 )$ as ${U_{p{\mathrm{ho}}}}( {0,t} )$. There’s also a probability that pSer2 at 3′ region of the genes does not increase as much as that at 5′ region. So $\tau ( t )$ was used to control the increase extend of pSer2 with $x$. Taken together, we have


\begin{eqnarray*}
{U_{p{\mathrm{ho}}}}\left( {x,t} \right) = U_{p{\mathrm{ho}}}^{ini}\left( {0,t} \right)\cdot U_{p{\mathrm{ho}}}^{el}\left( {x,t} \right),
\end{eqnarray*}

\begin{eqnarray*}
U_{p{\mathrm{ho}}}^{el}\left( {x,t} \right) = \tau \left( t \right)\left( {{e^{\theta \left( {x - \gamma } \right)}} + 1 - {e^{ - \theta \gamma }}} \right) + 1 - \tau \left( t \right).
\end{eqnarray*}

Where $1 - \tau ( t )$ is used to control that $U_{p{\mathrm{ho}}}^{el}( {0,t} ) \equiv 1$. If pSer2 state increases at 3′ region of the genes is weaker than that at 5′ region, $\tau ( t )$ would be <1.

In process 3, based on the previous knowledge that the paused Pol II have a high turnover at TSSs [50, 51] and will eventually drop out at termination sites, with less probability of dropping out at gene body regions, we hypothesized a two-mixture model to describe the probability of drop out along the gene.


\begin{eqnarray*}
{p_{ev}}\left( {x,t} \right) \sim \alpha \left( t \right){\mathrm{Exp}}\left( \lambda \right) + \left( {1 - \alpha \left( t \right)} \right)N\left( {\mu ,{\sigma ^2}} \right).
\end{eqnarray*}

Because both the low speed and the imperfect processivity could explain the promoter-proximal peak [52], for simplicity we blamed all the change in the amplitude of the peak to on the velocity change, which is enough to reproduce experimental results.

In process 4, given an initial amount of initiated Pol II and the number of Pol II in the effective pool, at regular interval we count the number of Pol II that dissociates from and loads onto the chromatin. For the Pol II that dissociates from the chromatin, the probability that the Pol II can be dephosphorylated and return to the effective Pol II pool is described by ${r_{\mathrm{depho}}}( t )$, which means ${r_{\mathrm{depho}}}( t )$ percent of the Pol II can return to the pool. The effective Pol II pool size is continuously updated by the current pool size minus the relative number of Pol II that initiates, plus the portion of the dropped Pol II that was dephosphorylated. The initiation frequency is also continuously updated according to the effective pool size.


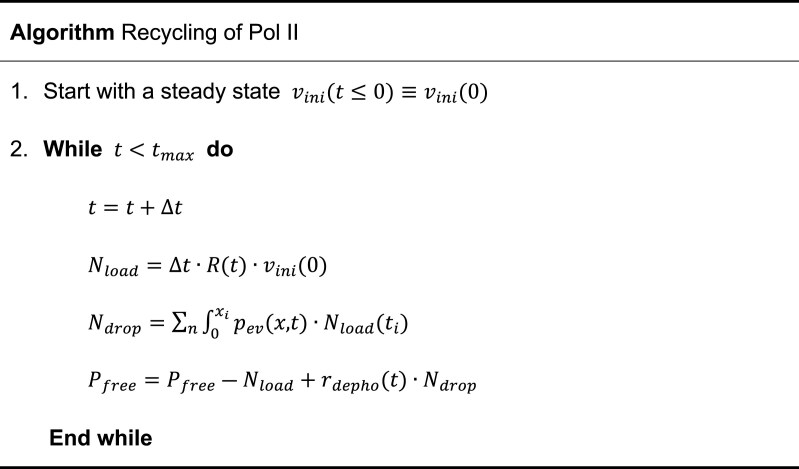


Where ${v_{\mathrm{ ini}}}( 0 )$ is the initial initiation frequency, ${N_{\mathrm{load}}}$ indicates the number of Pol II loading on to the gene, and is updated according to the change of effective pool size ${P_{\mathrm{free}}}( t )$. Considering that when the pool is large enough, the initiation frequency would not respond to the change of pool size, we set a threshold $Ts$ of the response.


\begin{eqnarray*}
R\left( t \right) = \left\{ {\begin{array}{@{}*{2}{c}@{}} {1,}&{{P_{\mathrm{free}}}\left( t \right) \ge Ts}\\ {\frac{{{P_{\mathrm{free}}}\left( t \right)}}{{Ts}},}&{{P_{\mathrm{free}}}\left( t \right) < Ts} \end{array}} \right..
\end{eqnarray*}

As mentioned earlier, the recycling factor was defined as the effective pool size normalized by the initial effective pool size ${P_{\mathrm{free}}}( t )/{P_{\mathrm{free}}}( 0 )$. The density of active Pol II on the gene is calculated as the proportion of Pol II remaining on the gene when an initial amount of Pol II extends to a certain position in the gene body, normalized by the interval between the two Pol II. Given a series of Pol II on the gene at position ${x_i}$ that loads on to the gene at time ${t_i}$, the active Pol II signal $A{P_c}( {{x_i},t} )$ is


\begin{eqnarray*}
A{P_c}\left( {{x_i},t} \right) = \left[ {1 - \int\limits_0^{{x_i}} {{p_{ev}}\left( {{x_i},t} \right)} } \right] \cdot \frac{{{N_{\mathrm{load}}}\left( {{t_i}} \right)}}{{{\Delta _x}}}.
\end{eqnarray*}

Where $\Delta x = {x_i} - {x_{i - 1}},{x_0} = 0$. Finally, the pSer2 state signals at any given time ${P_c}( {{x_i},t} )$, which is similar to pSer2 ChIP-Seq signal, is the product of pSer2 state per Pol II and the active Pol II signals.


\begin{eqnarray*}
{P_c}\left( {{x_i},t} \right) = {U_{pho}}\left( {{x_i},t} \right)\cdot A{P_c}\left( {{x_i},t} \right).
\end{eqnarray*}

${P_c}( {{x_i},t} )$ was normalized in order to compare with the real signal. And the real pSer2 ChIP-seq signals were counted as the reads normalized by spike-in in 500 bp bins.


\begin{eqnarray*}
{P_c}\left( {{x_i},t} \right){\mathrm{^{\prime}}} = {P_c}\left( {{x_i},t} \right)\cdot\frac{{\widehat {{P_c}\left( {{x_1},0} \right)}}}{{{P_c}\left( {{x_1},0} \right)}}.
\end{eqnarray*}

## Results

### CTDP1 regulates an intermediate phosphorylated state of Pol II (II-I)

**Figure 1.**
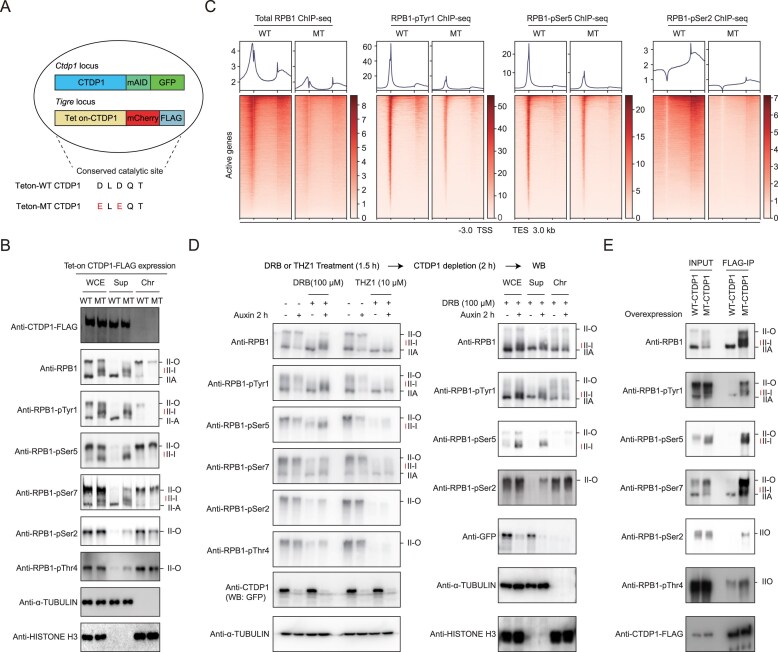
CTDP1 regulates a specific phosphorylation intermediate of Pol II. (**A**) Schematic of the strategy for exogenous inducible expression of WT or mutant (MT, D188/190E) CTDP1 at the *Tigre* locus in CTDP1 degron mESCs. (**B**) Western blot analysis of phosphorylated RPB1 forms in subcellular fractions of mESCs expressing exogenous WT or MT CTDP1. Histone H3 and α-Tubulin are shown as loading controls. (**C**) Meta-gene plots (top) and heatmaps (bottom) showing the changes in total RPB1, RPB1-pTyr1, RPB1-pSer5, and RPB1-pSer2 ChIP-seq signals from 3 kb upstream of the TSS to 3 kb downstream of the TES following the expression of exogenous WT or MT CTDP1 in mESCs. (**D**) Western blot analysis of phosphorylated RPB1 forms after drug treatment. Left: CTDP1 degron mESCs were treated with transcription inhibitors (DRB or THZ1) or vehicle control for 1.5 h prior to the addition or omission of 500 μM auxin for 2 h. Right: Subcellular fractions of DRB-treated cells. (**E**) Co-immunoprecipitation (Co-IP) of WT and MT CTDP1 in mESCs. Lysates from cells expressing FLAG-tagged WT or MT CTDP1 were immunoprecipitated with an anti-FLAG antibody, followed by immunoblotting with antibodies specific for distinct phosphorylated forms of RPB1. The input lane contains 5% of the total lysate used for immunoprecipitation.

Our previous work demonstrated that the Pol II subunit RPB7 recruits CTDP1 to regulate Pol II protein stability and transcription reinitiation [26]. To specifically investigate the dephosphorylation-related activity of CTDP1, we established stable cell lines expressing either WT or a phosphatase-deficient mutant of CTDP1 (D188/190E) in a CTDP1-degradable background (Fig. 1A and Supplementary Fig. S1A). These lines allowed us to compare WT and mutant versions at comparable expression levels. We further confirmed the lack of enzymatic activity in the CTDP1 mutants using an *in vitro* phosphatase assay with purified proteins (Supplementary Fig. S1B). We then performed Western blot analyses on cytoplasmic, nucleoplasmic, and chromatin fractions using antibodies against different phosphorylation states of Pol II. Surprisingly, we detected a distinct intermediate band between the classical II-A and II-O states, which was specifically enriched in the nucleoplasmic fraction. We termed this novel intermediate state “II-I” and confirmed that it is recognized by antibodies against Tyr1, Ser5, and Ser7 phosphorylation, but is weakly recognized by anti-Ser2 or anti-Thr4 antibodies (Fig. 1B). Additionally, we generated and evaluated the CTDP1 D188/190A mutant, observing that the II-I form was similarly present upon its expression (Supplementary Fig. S1C).

Subsequently, we conducted ChIP-seq experiments in CTDP1 WT and mutant cells using antibodies against total Pol II, pTyr1, pSer2, and pSer5. The enrichment of signals at expected genomic regions validated the efficacy of our ChIP-seq assays. We found that the CTDP1 mutant led to a global decrease in all examined phospho-forms of chromatin-associated Pol II (Fig. 1C and Supplementary Table S1). This result suggests that the accumulation of the II-I form in the nucleoplasm may represent a transcription intermediate complex whose prevalence was previously underappreciated. The increase in the abundance of the II-I phosphoform would reduce the pool of transcriptionally competent Pol II (pSer2 state).

To further elucidate CTDP1’s role in regulating Pol II dephosphorylation, we treated cells with the Pol II kinase inhibitors DRB and THZ1. DRB treatment with CTDP1 depletion, but not THZ1, led to an increase in the II-I level, which was also predominantly localized to the nucleoplasm (Fig. 1D). Furthermore, Co-IP assays revealed that the CTDP1 mutant exhibits a stronger interaction with the II-I form compared to the WT CTDP1 (Fig. 1E). This preferential binding likely reflects impaired phosphatase activity, resulting in slower dephosphorylation kinetics and a dominant-negative effect.

### CTDP1 specifically regulates the Pol II phosphorylation intermediate

**Figure 2.**
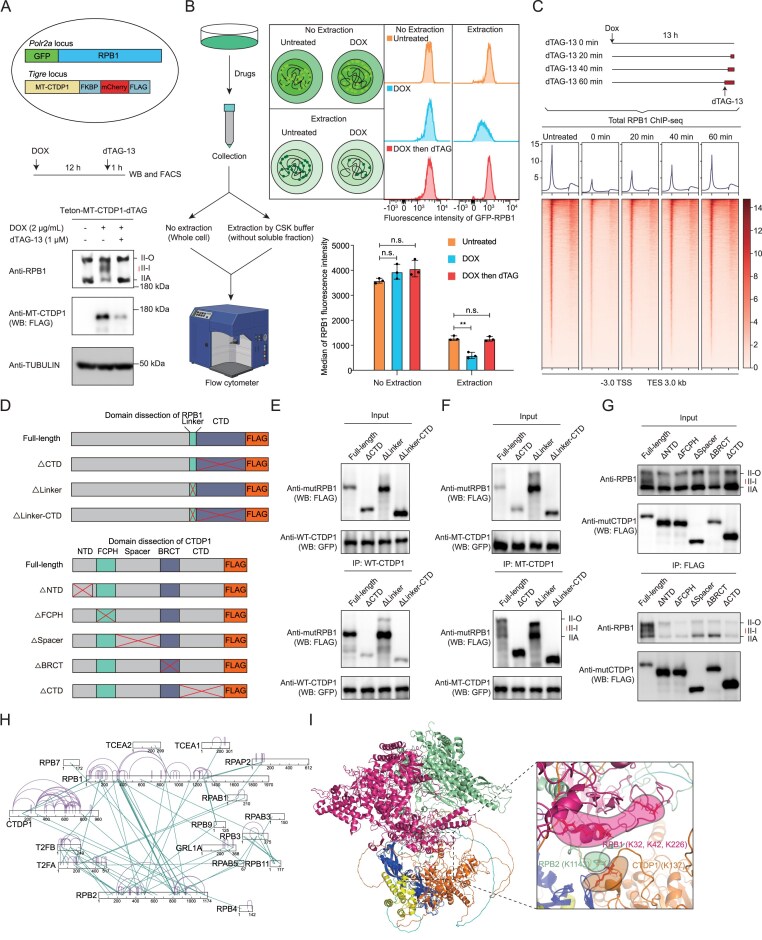
CTDP1 specifically regulates the II-I form of Pol II. (**A**) Top: Schematic of the strategy for inducible expression of degradable MT-CTDP1 (with FKBP degron) at the *Tigre* locus in GFP-RPB1 mESCs. Bottom: Western blot analysis of sequential treatment with DOX and dTAG-13. Cell lysates were prepared from untreated, DOX-only (13 h), and DOX (12 h) plus dTAG-13 (1 h) treated groups, respectively. α-Tubulin served as a loading control. (**B**) Left: Schematic for flow cytometric analysis of chromatin-bound GFP-RPB1 by cell permeabilization with CSK buffer (25 mM HEPES pH 7.4, 50 mM NaCl, 1 mM EDTA, 3 mM MgCl_2_, 300 mM sucrose, 0.5% Triton X-100, and protease inhibitor). Right: Distribution (top) and median (bottom, means ± SD) of fluorescence intensity under three different drug treatments with or without extraction treatment. Statistical significance, two-sided unpaired *t*-test, **P value < .01. (**C**) Time-course analysis of RPB1 ChIP-seq signals upon MT-CTDP1 degradation.​MT-CTDP1-dTAG mESCs were pretreated with Dox for 13 h, followed by the addition of dTAG-13 for the final 0, 20, 40, or 60 min (top). Meta-gene plots (middle) and heatmaps (bottom) show the changes in RPB1 ChIP-seq signals from 3 kb upstream of the TSS to 3 kb downstream of the TES. (**D**) Schematic of RPB1 and CTDP1 domain dissection constructs. Diagram illustrating the full-length and various domain dissection of RPB1 (top) and CTDP1 (bottom). For RPB1, deletions include ΔCTD, ΔLinker, and ΔLinker-CTD. For CTDP1, deletions include ΔNTD, ΔFCPH, ΔSpacer, ΔBRCT, and ΔCTD. (**E, F**) Co-IP analysis of CTDP1 (WT, left; MT, right) interaction with mutated RPB1 (mutRPB1) using Strep-Tactin beads in HEK293F. All RPB1 domain-dissection constructs with FLAG tag were transiently transfected into Twin-Strep-tagged WT or MT CTDP1 expressing cells. (**G**) Co-IP analysis of MT CTDP1-RPB1 interaction with five MT-CTDP1 truncation constructs (ΔNTD, ΔFCPH, ΔSpacer, ΔBRCT, and ΔCTD) in HEK293F. FLAG-tagged constructs of MT-CTDP1 domain-dissection mutants were transiently transfected and immunoprecipitated with an anti-FLAG antibody. (**H**) Overview of BS3 crosslinks in the CTDP1-Pol II complex from HEK293F. Green and purple lines depict inter- and intra-subunit crosslinks, respectively. (see Supplementary Data 3 for more details). (**I**) Crosslinks between CTDP1 and Pol II subunits (RPB1, RPB2) mapped onto the AlphaFold-predicted structure of the RPB7-50 × GS-CTDP1 protein, with involved lysines (K) annotated.

We next established a conditional expression system by introducing a doxycycline (dox)-inducible dTAG-CTDP1 mutant construct. In this system, the addition of dox induces expression of the CTDP1 mutant, while subsequent treatment with dTAG-13 triggers acute degradation of the mutant protein. Western blot analysis revealed that expression of the CTDP1 mutant led to increased levels of II-I Pol II. Conversely, degradation of the mutant CTDP1 specifically reduced II-I abundance and restored total Pol II levels (Fig. 2A). To examine chromatin-associated Pol II, we used 0.5% Triton X-100 lysis buffer to remove soluble nuclear fractions. Consistent with the nucleoplasmic accumulation of the II-I form, fluorescence-activated cell sorting (FACS) analysis revealed a decrease in chromatin-bound Pol II upon expression of mutant CTDP1 (Fig. 2B), indicating that CTDP1 regulates the nucleoplasmic pool of II-I Pol II. To gain molecular insights into the resumption of Pol II kinetics and its impact on gene regulation, we performed total Pol II ChIP-seq under the same experimental conditions used for FACS analysis. The results demonstrated a gradual recovery of Pol II occupancy following degradation of mutant CTDP1 across multiple time points (Fig. 2C). Subclassification of genes based on Pol II recovery kinetics revealed four distinct clusters. Clusters 1–3, which exhibited faster recovery, were enriched for RNA and DNA metabolic processes and showed higher levels of the active chromatin marks H3K27ac and H3K4me3. In contrast, Cluster 4, with the slowest recovery rate, was less transcriptionally active and enriched for genes involved in glucose catabolic processes (Supplementary Fig. S1D–F).

We next sought to identify the domain requirements for CTDP1-mediated regulation of II-I. First, we performed domain dissection of RPB1, the largest subunit of Pol II. Since previous studies indicated that most RPB1 phosphorylation occurs within its CTD and linker regions, we generated CTD-deleted and linker-deleted RPB1 variants. Our results showed that both CTD deletion and linker deletion abolished II-I signals. These effects were not observed with WT CTDP1 Co-IP (Fig. 2D–F). We then progressively deleted domains in mutant CTDP1 and found that nearly the entire CTDP1 protein was required for II-I appearance (Fig. 2G), suggesting that multiple domains contribute to its function.

To dissect the specific contributions of individual domains (e.g. FCPH and BRCT), we introduced point mutations into conserved motifs within the FCPH (D302K [53]) and BRCT (W710R [54]) domains (Supplementary Fig. S2A). We then expressed these mutants and assayed their ability to capture the II-I form. Notably, point mutations within either the FCPH or BRCT domain abolished CTDP1 binding to RPB1 and II-I form. Furthermore, we purified the mutant CTDP1 and performed cross-linking mass spectrometry, which confirmed direct interaction between CTDP1 and RPB1 (Fig. 2H and I; Supplementary Table S2). However, few peptides were detected from the Pol II CTD, likely due to the highly repetitive nature of this region and lack of trypsin digestion sites, which presents challenges for conventional mass spectrometric detection. Together, these findings demonstrate that the CTD and linker regions of RPB1 are required for CTDP1 to promote or stabilize the II-I form of Pol II. However, our interaction data indicate that MT-CTDP1 can associate with RPB1 even in the absence of these regions, suggesting that the regulation of the II-I form may involve additional core determinants or indirect scaffolding mechanisms. Further studies are needed to dissect the precise molecular basis of the CTDP1–Pol II interaction and its role in II-I formation.

Our Pol II ChIP-MS dataset revealed that the interaction between Pol II and the core subunits of PP2A was decreased under the MT-CTDP1 condition (Supplementary Fig. S2B), suggesting that MT-CTDP1 sterically hinders alternative phosphatases. Neither hyper-activating PP2A (via FTY720 treatment [55]) nor overexpressing the INTAC subunit INTS6 reduced the MT-CTDP1-associated II-I signal (Supplementary Fig. S2C). These results suggest that a simple phosphatase compensation model cannot account for the accumulation of the II-I form, indicating the involvement of a more complex kinetic interplay between multiple phosphatases and kinases [13, 56]. To directly demonstrate that the II-I form is indeed a phosphorylated species rather than a proteolytically cleaved byproduct of RPB1, we performed an *in vitro* dephosphorylation assay using Calf Intestinal Phosphatase (CIP). As shown in Supplementary Fig. S2D, the characteristic mobility shift of the II-I form is strictly dependent on reversible phosphorylation, and its complete conversion to the fully unmodified II-A form upon exogenous phosphatase treatment definitively establishes its identity as a dynamic phosphorylation intermediate.

### The phosphatase activity of CTDP1 is required for Mediator complex recruitment to Pol II

**Figure 3.**
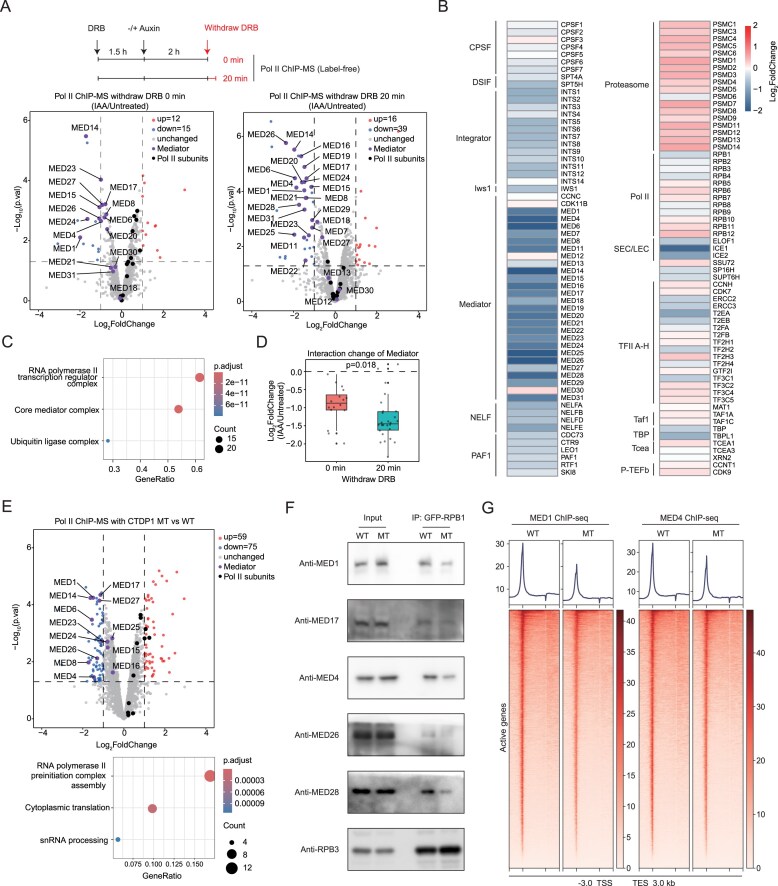
CTDP1 mediates the recruitment of the Mediator complex. (**A**) Schematic of the procedure of DRB treatment-and-release experimental design followed by Pol II ChIP-MS in CTDP1 degron mESCs. Volcano plot showing the protein enrichment changes identified by label-free MS at 0 min (left) and 20 min (right) after DRB release upon depletion of CTDP1. The purple dots indicate Mediator subunits and the black dots indicate Pol II subunits. The differentially enriched proteins were identified by DEP (|log_2_FC|>1, *P* value < .05, moderated *t*-test). (**B**) Heatmap of changes in protein enrichment of a subset of Pol II-associated factors in Pol II ChIP-MS 20 min after DRB release upon depletion of CTDP1. (**C**) GO term enrichment analysis of down-regulated proteins in Pol II ChIP-MS 20 min after DRB release upon depletion of CTDP1. (**D**) Boxplots showing the log_2_FC for Mediator subunits at DRB release 0 min and 20 min upon depletion of CTDP1. Statistical significance was determined by the Wilcoxon rank-sum test. (**E**) Top: Volcano plot showing protein enrichment changes in Pol II ChIP-MS comparing WT- and MT-CTDP1 expressing mESCs. The differentially enriched proteins were identified by DEP (|log_2_FC|>1, *P* value < .05, moderated *t*-test). Bottom: GO term enrichment analysis of down-regulated proteins in Pol II ChIP-MS. (**F**) Western blot analysis of GFP-RPB1 ChIP eluates quantified the enrichment levels of specific Mediator subunits in mESCs expressing WT or MT CTDP1. RPB3 was used as a loading control. (**G**) Meta-gene plots (top) and heatmaps (bottom) showing the changes in Mediator Complex Subunit 1 (MED1) and Mediator Complex Subunit 4 (MED4) ChIP-seq signals from 3 kb upstream of TSS to 3 kb downstream of the TES following overexpression of WT or MT CTDP1 in mESCs.

Our previous work established that a DRB treatment-and-release regimen enables analysis of Pol II transcription reinitiation dynamics [26]. Using this system, we induced Pol II reinitiation following DRB washout and specifically degraded CTDP1, followed by Pol II ChIP-MS. Upon 20 min of DRB release, CTDP1 depletion led to a marked decrease in Mediator complex components, but not under the 0-min washout condition (Fig. 3A and B), indicating that CTDP1-mediated defects in Pol II reinitiation involve impaired Mediator recruitment. We also observed an increase in proteasome subunits (Fig. 3B), consistent with previously reported Pol II instability upon CTDP1 loss [26]. Among the most enriched factors were Mediator components, whereas other Pol II-associated factors showed neither uniform nor consistent changes (Fig. 3C and D).

We next performed Pol II ChIP-MS in WT versus CTDP1 mutant mESCs. Concurrently, we observed reduced association of the Mediator complex with Pol II in the mutant cells (Fig. 3E). Furthermore, Co-IP and ChIP-seq experiments confirmed that the presence of MED1 and MED4 was decreased in Pol II immunoprecipitates and at chromatin occupancy sites, respectively (Fig. 3F and G). It is widely accepted that Mediator preferentially associates with the unmodified (hypophosphorylated) CTD of Pol II during PIC assembly [57–59]. Our results demonstrate that the dephosphorylation activity of CTDP1 is necessary for recruiting the Mediator complex to Pol II, providing a molecular mechanism for the role of CTDP1 in Pol II-mediated transcription regulation.

### CTDP1 plays a crucial role in Pol II transcription efficiency

**Figure 4.**
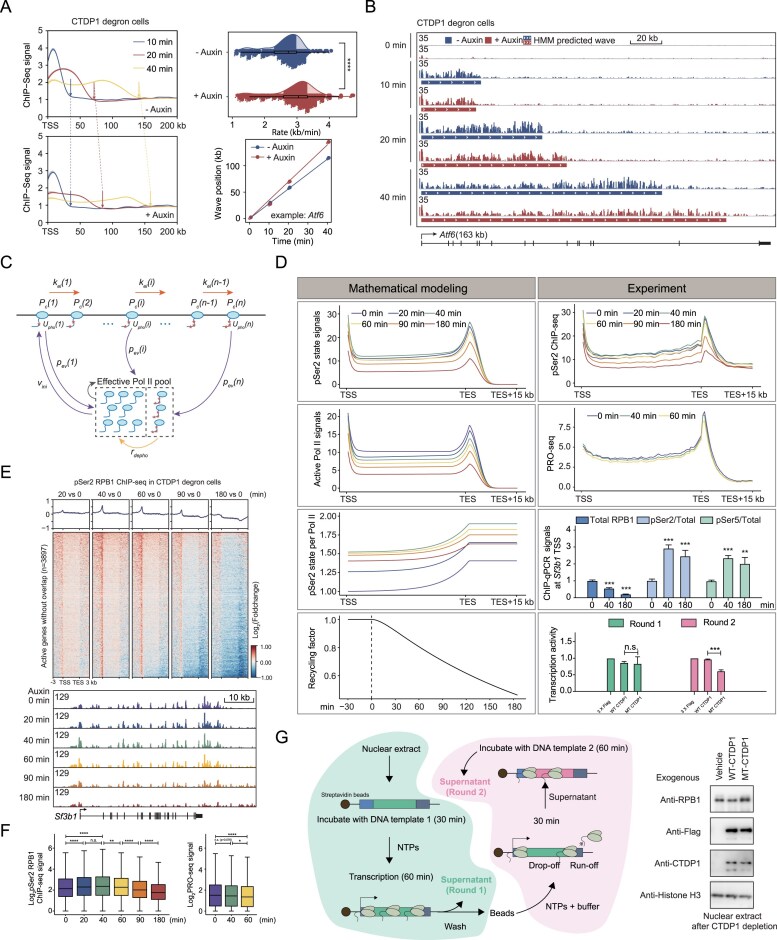
CTDP1 is crucial for Pol II transcription efficiency. (**A**) Left: Meta plots showing RPB1-pSer2 ChIP-seq signal of the long genes with predictable waves (*n* = 249) after washing out DRB for 10, 20, and 40 min upon the degradation of CTDP1 in mESCs. Right: Slab plots showing the mean velocity predicted by Hidden Markov Model (HMM) of the genes upon CTDP1 degradation. Below shows the measured wave and velocity of an example gene *Atf6*. (**B**) The snapshot of DRB/RPB1-pSer2 ChIP-seq for *Atf6* gene. The bars with arrows are the waves predicted by HMM. (**C**) A schematic diagram of the mathematical model (details see “Materials and methods” section). (**D**) Left shows RPB1-pSer2 state signals, active Pol II signals, pSer2 state per Pol II, and recycling factor predicted by mathematical simulation. Right shows meta-gene plots of RPB1-pSer2 ChIP-seq signal, meta-gene plots of PRO-seq signal, ChIP-qPCR signals at *Sf3b1* gene locus, and transcription activity measured by *in vitro* recycling assay. For qPCR assay, four biological replicates were performed. Statistical significance, independent *t*-test; ***P value < .001; *P value < .05; n.s., not significant; error bars, SD. For qPCR primers see Supplementary Table S4. (**E**) Heatmap showing the log_2_ fold change of RPB1-pSer2 ChIP-seq upon the degradation of CTDP1 after 20, 40, 60, 90, and 180 min versus 0 min. Bottom: Genome browser showing RPB1-pSer2 ChIP-seq tracks at *Sf3b1* gene locus upon CTDP1 degradation. (**F**) Boxplot showing the quantification of pSer2 ChIP-seq (left) or PRO-seq (right) signal at gene body region (+1 kb downstream of TSS to TES) upon the degradation of CTDP1. Statistics were performed using Wilcoxon test (n.s. *P* value > .05, * *P* value < .05, ** *P* value < .01, *** *P* value < .001, **** *P* value < .0001). (**G**) Left: Schematic of the procedure of *in vitro* transcription recycling assay [32]. Right: Western blot analyses in the CTDP1-degraded mESCs nuclear extract carrying three kinds of FLAG peptide eluting fractions (FLAG peptides, WT-CTDP1-Flag and MT-CTDP1-Flag).

To assess Pol II elongation upon CTDP1 loss, we performed pSer2-RPB1 ChIP-seq in CTDP1 degron cells at 0, 10, 20, and 40 min after DRB release, under CTDP1-depleted conditions. The results (Fig. 4A and B) showed an increased elongation rate, which served as the basis for the mathematical modeling described below. Due to the challenging nature of investigating Pol II II-I in cells, we employed mathematical modeling and experiments to explore the transcription process of Pol II. Our mathematical investigation was guided by three assumptions based on experimental results and previous *in vitro* assay with CTDP1 [26]: (a) CTDP1 degradation resulting in increased pSer2 signals. (b) CTDP1 degradation causing RPB1 destabilization. (c) CTDP1 depletion leading to an increased Pol II transcription rate, as indicated by our DRB inhibition release experiments in the absence of CTDP1 (Fig. 4A and B). The velocity measurement was obtained from a curated set of 249 genes (selection criteria detailed in the “Materials and methods” section), and the resulting model is not intended to be extrapolated as a universal genome-wide model. In the mathematical model, the Pol II initiates from the effective Pol II pool and elongates along the gene coupled by phosphorylation and dissociation events. The dissociating Pol II needs to be dephosphorylated to return to the pool and reinitiate. The model allows the generation of pSer2 state signals, active Pol II signals and pSer2 state per Pol II (Fig. 4C, detailed definition see “Materials and methods” section).

We were surprised to find that the incorporation of merely the three assumptions listed above was enough to produce an increase followed by a decrease pattern of the pSer2 occupancy across genes after the degradation of CTDP1 at different time points (Fig. 4D, line 1). Indeed, our pSer2 ChIP-seq after CTDP1 degradation at different time points corroborated this trend (Fig. 4E and F, left panel). Our mathematical modeling also forecasted the decrease of the number of active Pol II during CTDP1 degradation. Our CTDP1 depletion followed by PRO-seq data aligns with this prediction (Fig. 4D, line 2; 4F, right panel). Consistently, the model predicted an initial increase in the pSer2 state per Pol II, followed by a decrease after a prolonged depletion of CTDP1. Our ChIP-qPCR results also showed a similar trend (Fig. 4D, line 3). The validation of our mathematical modeling through the aforementioned experiments led us to predict the recycling ability of Pol II using the normalized effective Pol II pool size, which indicates the number of Pol II in the pool that was able to recycle (recycling factor, detailed definition see “Materials and methods” section). The recycling factor is predicted to gradually decrease after the loss of CTDP1 (Fig. 4D, line 4). Subsequently, we conducted previously established *in vitro* Pol II recycling assays using purified WT and mutant CTDP1 [32]. The results demonstrated that CTDP1 mutant led to decreased Pol II recycling activities *in vitro* (Fig. 4D, line 4; 4G). Collectively, these findings establish CTDP1 as a key regulator of the functional Pol II pool by controlling its recycling between termination and reinitiation.

### The phosphatase activity of CTDP1 is critical for regulating RNA splicing

**Figure 5.**
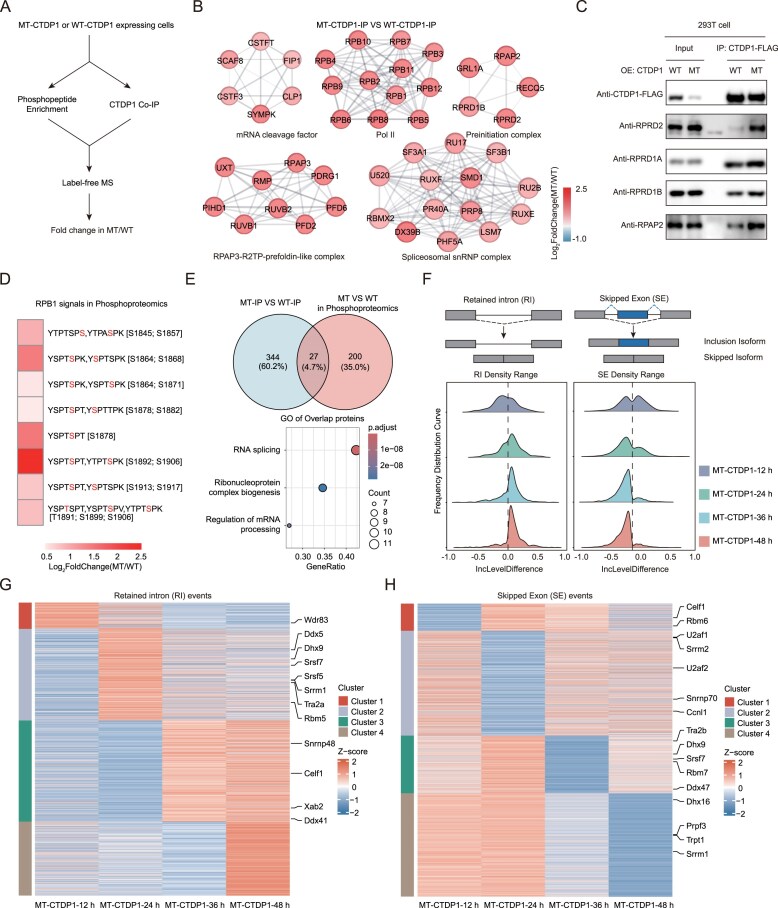
CTDP1 activity modulates RNA splicing. (**A**) Schematic overview of the CTDP1 interactome and phosphoproteomics strategy. MT-CTDP1 or WT-CTDP1-expressing mESCs were subjected to whole-cell phosphopeptide enrichment and CTDP1 Co-IP, followed by MS analysis. (**B**) A network showing the protein complexes enriched in CTDP1 IP-MS. The color of the circles indicates the log_2_FC(MT/WT) enrichment score in CTDP1 IP-MS. (**C**) Following transfection of HEK293T cells with FLAG-tagged WT- or MT-CTDP1, Co-IP with an anti-FLAG antibody was performed to assess CTDP1 interactions with transcriptional regulatory factors. Input lysates (5%) and immunoprecipitates were immunoblotted with antibodies against the indicated proteins, using CTDP1-FLAG as a loading control. (**D**) Heatmap showing phosphorylation changes of heptapeptide repeats of RPB1. Each row corresponds to a phosphopeptide identified in the phosphoproteomic analysis, with specific modified residues highlighted and their positions on RPB1 indicated. (**E**) Top: Venn diagram showing the intersection of proteins that were significantly up-regulated in CTDP1 IP-MS and in whole-cell phosphoproteomic profiling comparing WT CTDP1 and MT CTDP1 cells. Bottom: GO term enrichment analysis of overlapping proteins in Venn diagram. (**F**) Top: Diagram illustrating two types of alternative splicing events: RI and SE. For each event, both the inclusion isoform and the skipped isoform are shown, demonstrating the alternative transcript structures quantified in the RNA-seq analysis. Bottom: Density plots showing the frequency distribution of IncLevelDifference values for RI and SE events at each time point after expressing MT-CTDP1 in mESCs. IncLevelDifference is defined as the inclusion level at a given time point minus the inclusion level at 0 h. Curves represent the smoothed frequency distribution of splicing changes, illustrating the global shifts in exon inclusion or intron retention across the time course. (**G**) Heatmap showing hierarchical clustering of IncLevelDifference values for RI events across the time course. For each RI event, IncLevelDifference was scaled by row-wise *Z*-score. RNA-splicing-related genes associated with the RI clusters are annotated on the right. (**H**) Heatmap showing hierarchical clustering of IncLevelDifference values for SE events across the time course. SE events were processed and scaled using the same workflow as in panel (G), and RNA-splicing-related genes associated with SE clusters are similarly annotated.

To obtain a global view of CTDP1-dependent dephosphorylation substrates, we performed immunoprecipitation coupled with mass spectrometry (IP-MS) using CTDP1 WT and phosphatase-deficient mutant cells, along with corresponding whole-cell phosphoproteomic analyses (Fig. 5A and Supplementary Table S2). The IP-MS results revealed that the CTDP1 mutant exhibited enhanced interactions with Pol II (Supplementary Fig. S2E–G) and pre-initiation complexes (Fig. 5B), consistent with our earlier observations. Interestingly, we also detected increased association between the CTDP1 mutant and components of the RNA splicing machinery, RNA cleavage complexes, and the R2TP chaperone complex (Fig. 5B). A subset of these interactions was independently validated by Co-IP and western blotting (Fig. 5C).

Phosphoproteomic profiling revealed elevated phosphorylation of the Pol II CTD at serine residues in CTDP1 mutant cells, with a pronounced increase specifically at serine 5 within the conserved heptad repeat motif (Fig. 5D and Supplementary Fig. S2H). However, due to the highly repetitive sequence of CTD peptides, complete site-specific assignment remained challenging. To identify more direct substrates of CTDP1, we integrated the interaction data with phosphoproteomic changes. While the overlap between interaction-increased and phosphorylation-increased proteins was limited—potentially due to stringent thresholds or binary classification—the overlapping proteins were significantly enriched for factors involved in RNA processing (Fig. 5E and Supplementary Fig. S2I). To validate direct substrates, we performed *in vitro* dephosphorylation assays using purified recombinant WT-CTDP1 and synthetic phosphopeptides (Supplementary Fig. S2J). CTDP1 robustly dephosphorylated the positive controls (RPB1-CTD-pSer2 and RPB1-CTD-pSer5) and, importantly, directly dephosphorylated candidate phosphosites on the splicing factors RBM14, YTDC1, and BCLF1. These results demonstrate that CTDP1 directly acts on multiple splicing-regulatory proteins, strengthening the mechanistic link between its phosphatase activity and the observed splicing alterations.

**Figure 6.**
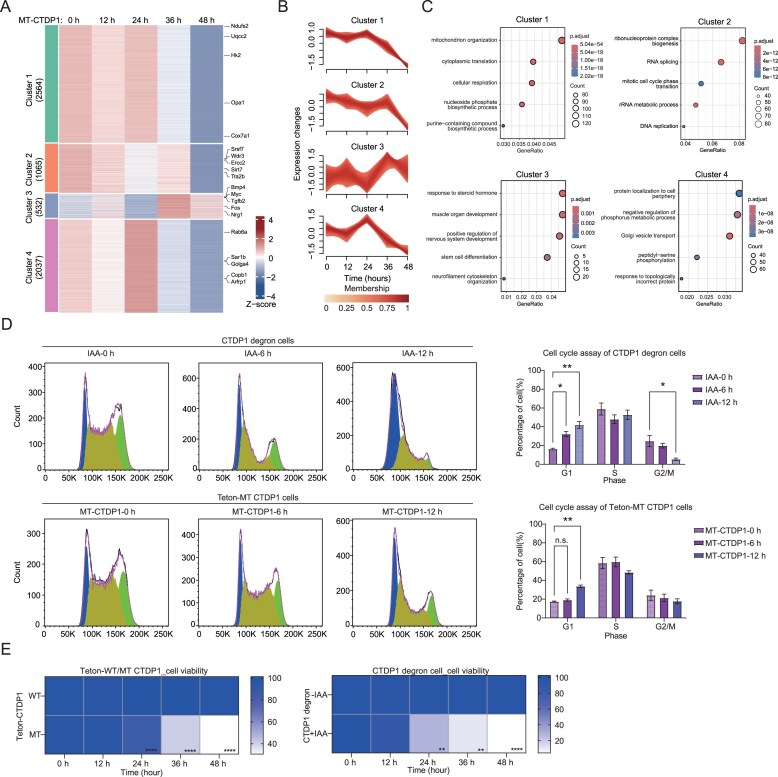
CTDP1 is a key regulator of oscillatory gene expression, cell cycle progression, and cell viability. (**A**) Heatmap showing time-course gene expression gene pattern derived from spike-in-normalized RNA-seq counts, which were scaled by row-wise *Z*-score. Fuzzy c-means clustering was performed using R package Mfuzz, yielding four major temporal expression clusters. Only genes with a membership score >0.6 within their assigned cluster were selected into further analysis. Representative genes from significantly enriched GO terms are annotated on the right. (**B**) Time-course expression patterns of spike-in-normalized genes were clustered into four groups using Mfuzz. The four resulting clusters are shown with their standardized expression changes across the 0–48 h time course. The color of the curves indicates the membership score for a specific cluster. (**C**) GO term enrichment analysis of each of the four temporal expression clusters identified from Mfuzz. (**D**) Flow cytometry of CTDP1 degron and Teton-MT CTDP1 mESCs treated with IAA or Dox for 0, 6, and 12 h, respectively. Left, representative DNA content histograms (PI staining). Right, quantification of cell cycle phases. Statistics, three biological replicates, mean ± SD. *P* values, two-way ANOVA with Tukey’s multiple comparisons test. (**E**) Cell proliferation of Teton-WT/MT CTDP1 and CTDP1 degron mESCs treated with IAA or Dox for 0, 12, 24, 36, and 48 h, respectively. CCK–8 assays showing the viability of Teton–WT/MT CTDP1 cells (left) and CTDP1 degron cells (right) treated for the indicated times. Statistics, three biological replicates, mean ± SD. *P* values, two-way ANOVA with Tukey’s multiple comparisons test.

We further analyzed RNA splicing outcomes using a time-series dataset of CTDP1 mutant expression in mESCs (Fig. 6A). Alternative splicing events—including alternative 3′ and 5′ splice sites, mutually exclusive exons, intron retention, and cassette exons—were systematically examined. Splicing analysis demonstrated that intron retention significantly increased, while cassette exon inclusion decreased, in a time-dependent manner upon induction of the CTDP1 mutant (Fig. 5F). The number of identified alternative 3′ and 5′ splice sites and mutually exclusive exon events is substantially lower than that of SE and RI (Supplementary Table S3). Furthermore, the overall magnitude of change for these three additional event types was not as pronounced across the treatment time points (Supplementary Fig. S2K). Heatmap visualization of individual splicing events revealed dynamic and temporally distinct patterns of intron retention and exon inclusion. Notably, many of the affected splicing events involved genes encoding splicing regulators themselves, suggesting a potential feedback mechanism (Fig. 5G and H). Together, these results establish that the phosphatase activity of CTDP1 plays a critical role in regulating RNA splicing, potentially through modulating the phosphorylation status of and functional interactions with splicing complexes.

We next performed a parallel overlap analysis for alternative splicing events, focusing specifically on significantly increased SE and RI events between the CTDP1 mutant and CTDP1 depletion conditions (Supplementary Fig. S2L). For this analysis, the overlap ratio was calculated using the total number of MT-CTDP1-induced AS events at each time point as the denominator. This overlap ratio increased progressively across the MT-CTDP1 time course. We also quantified the fraction of CTDP1-degron down-regulated genes that were concurrently down-regulated by MT-CTDP1 expression at each time point. This transcriptomic overlap ratio also increased steadily over time. Interestingly, the overlap ratio for differentially spliced genes was notably lower than that observed for differentially expressed genes. This divergence suggests that certain splicing alterations may be regulated through dephosphorylation-independent mechanisms, or alternatively, may simply reflect the inherently limited dynamic range of splicing changes relative to global transcriptional alterations.

### CTDP1 regulates cell cycle progression

Given the critical role of CTDP1 in regulating Pol II II-I dephosphorylation, transcription efficiency, and RNA splicing, we sought to investigate its broader biological functions in mESCs. Using time-series RNA-seq data following the induction of CTDP1 mutation, we identified four distinct patterns of gene expression dynamics (Fig. 6A and Supplementary Table S3). All clusters exhibited similar down-then-up expression trajectories, although the timing of these shifts​ varied (Fig. 6B). Cluster 1 exhibited an initial decrease, followed by an increase and a subsequent decrease. Genes in this cluster were enriched in mitochondrial organization and cytoplasmic translation. Cluster 2 showed an initial decrease, a slight increase, and then a decrease, with enrichment in ribonucleoprotein complex biogenesis, cell cycle control, and RNA splicing. Cluster 3 displayed an increase, decrease, increase, and decrease pattern, and was enriched in steroid hormone response and muscle organ development. Cluster 4 demonstrated a decrease-increase-decrease trajectory, with genes involved in protein localization to the cell periphery and phosphorus metabolic processes (Fig. 6C). We next examined the effects of CTDP1 depletion or mutation on cell cycle distribution. Degradation of CTDP1 or expression of the CTDP1 mutant resulted in an increased proportion of cells in G1 phase, indicating that CTDP1 is involved in cell cycle regulation (Fig. 6D), consistent with previous literature highlighting CTDP1’s role in cell cycle regulation [60–62]. Furthermore, loss of functional CTDP1 led to a significant decrease in cell viability, underscoring its importance in maintaining cellular homeostasis (Fig. 6E). Together, these findings position CTDP1 as a key regulator of cell cycle progression and cellular viability, with potential implications for cell proliferation control.

## Discussion

The phosphorylation of Pol II, transitioning from the hypo-phosphorylated II-A state to the hyper-phosphorylated II-O state, is a tightly regulated process central to transcription. A long-standing question in the field has been whether the hyper-phosphorylated Pol II is directly dephosphorylated to an unphosphorylated form or transitions through an intermediate phosphorylation state. In this study, we employed a CTDP1 mutant and identified a distinct intermediate Pol II state (designated II-I), which predominantly localizes to the nucleoplasm and is phosphorylated at tyrosine 1 (Tyr1), serine 5 (Ser5), and serine 7 (Ser7), as detected using established Pol II phospho-specific antibodies. While the identification of the II-I state advances our understanding of Pol II phosphorylation dynamics, it also raises several new questions. These include elucidating the mechanism of transition from the II-I to the II-A state, developing methods to specifically capture the II-I form, defining its functional roles, understanding its regulatory mechanisms, and determining its contributions to development and disease. Additionally, direct biophysical validation of the D188/190E mutant’s folded state warrants future investigation to fully interpret the structural underpinnings of our observations.

The relationship between our identified II-I state and the recently described RNAPII-PP (IIPP) is critical for accurately positioning our findings within the transcription field. Our cellular fractionation experiments demonstrate that the II-I state predominantly localizes to the soluble fraction, whereas the IIPP state has been reported as a paused Pol II species tightly associated with chromatin (as shown in Fig. 2E of [63]). Given that the IIPP state accumulates under DRB treatment combined with INTS6 knockdown, we investigated whether overexpressing INTS6 could resolve the II-I form induced by MT-CTDP1 expression. However, under the conditions tested, overexpression of INTS6 exerted no detectable effect on the abundance of the II-I form. While our current data do not completely define the precise structural relationship between II-I and IIPP, both phenomena can be reconciled by a unified Kinetic Trapping Model (Supplementary Fig. S2M): catalytically inactive MT-CTDP1 stably binds Pol II and shields the substrate, physically preventing alternative phosphatases (such as INTAC/PP2A) from accessing the target sites, thereby trapping a normally transient intermediate through steric hindrance. Concurrently, DRB treatment stalls the forward kinase pathway, preventing the transition from this intermediate state to the hyper-phosphorylated II-O state, while CTDP1 depletion or INTS6 knockdown paralyzes the backward dephosphorylation pathway. Trapped between these blocked forward and backward transitions, the intermediate state inevitably accumulates due to this kinetic bottleneck. Ultimately, the II-I form represents a phosphorylation intermediate captured under specific kinetic constraints, and its broader physiological regulatory functions—beyond acting as a transient passenger in the dephosphorylation cascade—remain to be established in future investigations. Although the II-I isoform is currently captured under specific kinetic bottlenecks, its remarkably low steady-state level under physiological conditions implies that Pol II dephosphorylation is a highly orchestrated, stepwise, and finely tuned regulatory process, rather than a single-step, uncoordinated reaction.

Our results are consistent with the classical model, as CTDP1 inactivation reduces chromatin–associated Pol II and increases phosphorylated forms [26, 64–68]. Our study further demonstrates that the phosphatase activity of CTDP1 is critical for Mediator complex recruitment, transcription efficiency, and RNA splicing. However, it remains unclear whether these functions depend solely on CTDP1’s activity toward the Pol II II-I state or also involve dephosphorylation of other CTDP1 substrates. The endogenous II-I Pol II is typically undetectable by standard western blotting and cellular fractionation, suggesting that the CTDP1-mediated II-I state is highly transient and rapidly converts to the II-A form. Furthermore, generating specific mutations to study the II-I state is challenging due to the repetitive nature of the Pol II CTD and the unresolved specificity of its phosphorylation patterns, not to mention investigating the direct functions of II-I under physiological conditions. Additionally, the specific perturbations in intron retention and cassette exon splicing, along with the time-dependent alterations in splicing events, imply a potential role for CTDP1 in regulating splicing dynamics or modulating dephosphorylation cascades within splicing networks.

## Supplementary Material

gkag772_Supplemental_Files

## Data Availability

The datasets generated and analyzed during the current study are available in public repositories. The sequencing data are available in the GEO repository under accession number GSE223475 and GSE312502. The proteomics data are available in the ProteomeXchange repository with identifier PXD071392. The source code for mathematical modeling is available at https://github.com/xuqiqin/c1model.
