# Supplementary material for: CTDP1 governs a Pol II phosphorylation intermediate and coordinates Mediator recruitment, transcription efficiency, and splicing
Source: Nucleic Acids Res. 2026 Aug 6;54(14):gkag772. doi: 10.1093/nar/gkag772 (PMC13443452; doi:10.1093/nar/gkag772)
Supplement: Supplementary file 1 [file gkag772_supplemental_files.zip › Supplementary_Figure-20260801.pdf]

Supplementary Figures

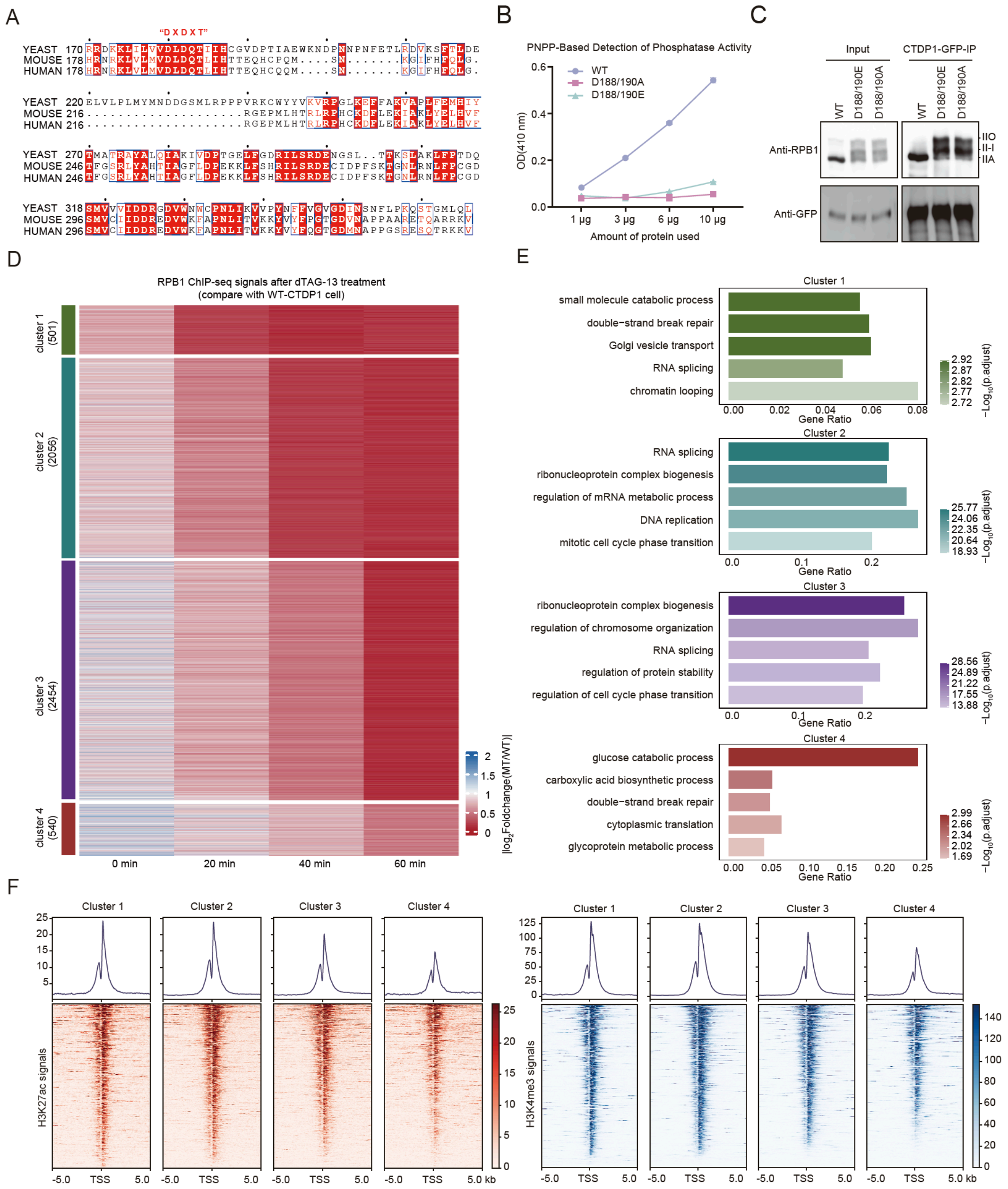

Figure S1 CTDP1 activity governs the dynamic control of Pol II chromatin binding.

A. Schematic of the multiple sequence alignment for the CTDP1 catalytic domain across yeast, mouse, and human. Strictly conserved and similar residues are denoted by white-on-red and red-on-white shading, respectively. The catalytic "DXDXT" motif is specifically labeled.

B. *In vitro* phosphatase assay of WT and mutant CTDP1. Enzymatic activity was measured using PNPP as a substrate, and product formation (p-nitrophenol) was quantified by absorbance at 410 nm. Data are represented as mean ± SD.

C. In HEK293T cells overexpressing the wild-type, D188/190E, and D188/190A CTDP1, co-immunoprecipitation (Co-IP) experiments confirmed that both mutant forms are able to bind to II-I. Whole cell lysates are shown as “Input”, while the “IP” fraction represents CTDP1-enriched samples.

D. Distinct transcriptional recovery kinetics and associated biological functions. The heatmap showing the absolute $\log_2$ fold change ($|\log_2 FC|$) in total RPB1 ChIP-seq signal in MT-CTDP1-dTAG cells relative to wild-type, categorized into four distinct clusters defined by their respective recovery times: 20 minutes, 40 minutes, 60 minutes, and the final recovery phase.

E. Gene Ontology (GO) enrichment analysis of biological processes significantly associated with genes in each clusters. Differentially cluster genes were identified using DESeq2 with a two-sided Wald test.

F. Distinct epigenetic landscapes associated with Pol II recovery kinetics. Top, meta-profiles of H3K27ac ChIP-seq signal around the TSS (± 5.0 kb) for the four Pol II recovery clusters. Bottom, profiles of H3K4me3 ChIP-seq signal across the same genomic region and clusters.

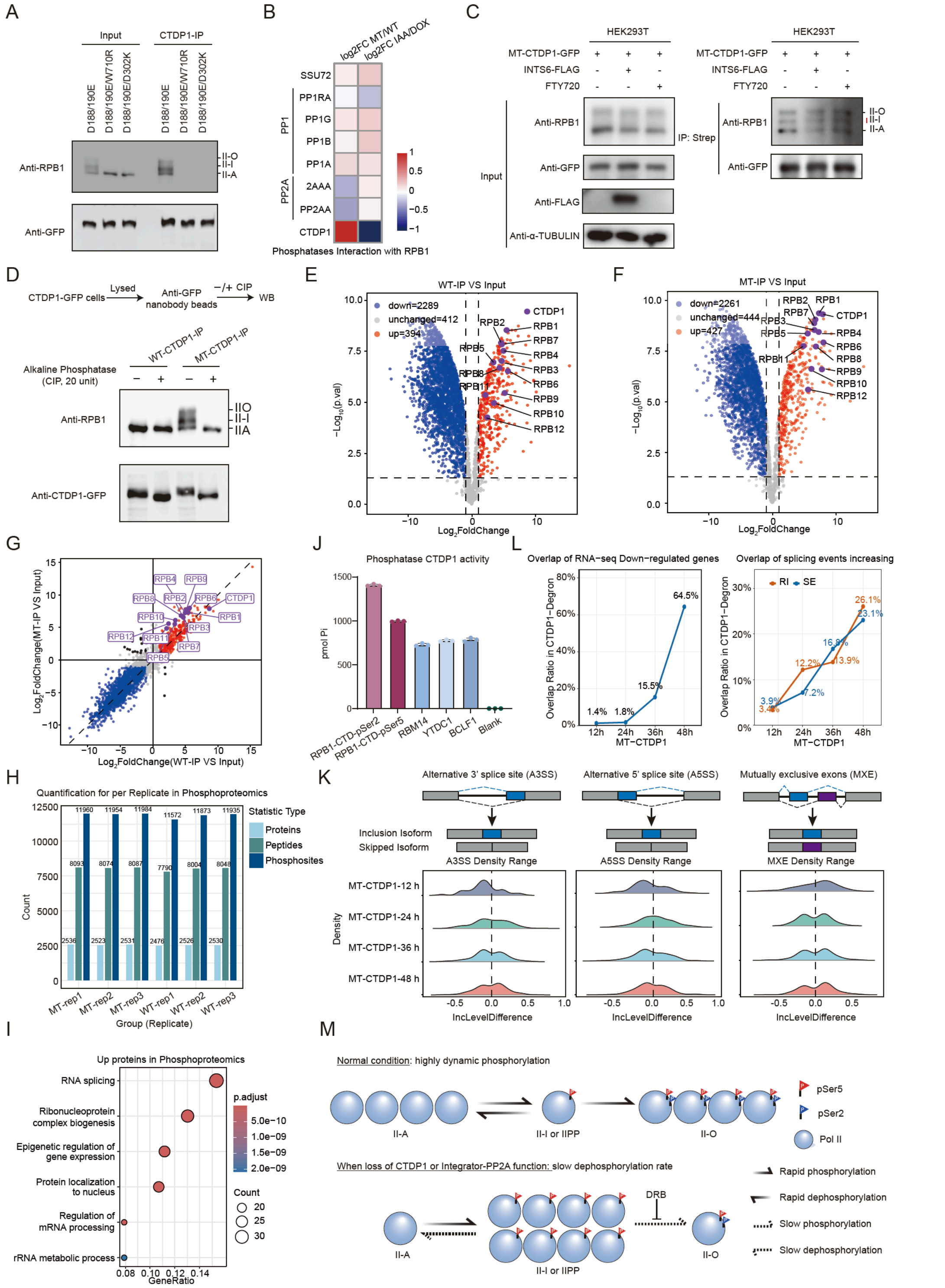

Figure S2 Proteomic and RNA analyses to define CTDP1 Function.

A. HEK293T cells overexpressing the indicated CTDP1 constructs were analyzed by Co-IP assay. “Input” denotes whole cell lysates; “IP” indicates CTDP1-enriched fractions.

B. The heatmap shows the differential interaction of selected phosphatases with RPB1. Interaction intensities were determined by RPB1 ChIP-MS (Label-free). The heatmap compares the enrichment of various phosphatase subunits in cells expressing MT-CTDP1 and cells undergoing CTDP1 depletion. Values represent the Log_2(fold change) of protein

abundance.

C. HEK293T cells expressing MT-CTDP1-GFP were either co-transfected with INTS6-FLAG or treated with FTY720 (25 μM, 1.5 h). Whole-cell lysates were analyzed by immunoblotting as “Input”. MT-CTDP1-GFP-associated complexes were enriched by Strep-tag immunoprecipitation as “IP” fraction.

D. The previously identified phosphorylated intermediate II-I was either untreated or treated with calf intestinal alkaline phosphatase (CIP) *in vitro*. The reaction products were separated by SDS-PAGE and analyzed by immunoblotting. CTDP1 signals served as a loading control.

E. Volcano plot showing protein enrichment changes in CTDP1 IP-MS comparing WT-CTDP1 IP fraction with the input control. The purple dots indicate Pol II subunits. The differentially enriched proteins were identified by DEP ($|\log_2 FC|>1$, p value < 0.05, moderated t-test).

F. Volcano plot showing protein enrichment changes in CTDP1 IP-MS comparing MT-CTDP1 IP fraction with the input control. The purple dots indicate Pol II subunits. Differential enrichment was assessed using the same criteria as in panel E.

G. Scatter plot showing the CTDP1 interactome. The x-axis represents $\log_2$FC of WT-CTDP1 IP versus input control, and the y-axis represents $\log_2$FC of MT-CTDP1 IP versus input control. Pol II subunits are highlighted.

H. Bar plot summarizing the number of identified proteins, phosphopeptides, and phosphorylation sites in the phosphoproteomic analysis of MT-CTDP1 and WT-CTDP1 cells (three biological replicates each).

I. GO term enrichment analysis of up-regulated proteins identified in the phosphoproteomic comparison of MT-CTDP1 versus WT-CTDP1 cells.

J. *In vitro* phosphatase assay using purified WT-CTDP1 and phosphopeptides as substrates. Phosphatase activity was detected by measuring free phosphate release with a malachite green detection kit. Data are represented as mean ± SD.

K. Top: Diagram illustrating three types of alternative splicing events: alternative 3' splice site (A3SS), alternative 5' splice site (A5SS) and mutually exclusive exons (MXE). For each event, both the inclusion isoform and the skipped isoform are shown, demonstrating the alternative transcript structures quantified in the RNA-seq analysis. Bottom: Density plots showing the frequency distribution of IncLevelDifference values for these events at each time point after expressing CTDP1 mutant. Curves represent the smoothed frequency distribution of splicing changes.

L. Left plot showing the proportion of CTDP1-degron down-regulated genes that were also down-regulated upon MT-CTDP1 expression at 12, 24, 36, and 48 h. Right plot showing the overlap of significantly increased splicing events between CTDP1-degron and MT-CTDP1 conditions. SE and RI events are shown separately.

M. A kinetic trapping model explaining the accumulation of the Pol II II-I phosphorylation state. Under physiological conditions, rapid kinase and phosphatase activities drive dynamic cycling between the II-A, II-I, and II-O states, maintaining the II-I form at a low, transient steady-state level. Complete depletion or dominant-negative inhibition of CTDP1 or the Integrator-PP2A complex severely decelerates dephosphorylation. Concurrently, DRB treatment blocks the forward kinase pathway, halting the transition from the II-I to the

hyper-phosphorylated II-O state. Caught within this kinetic bottleneck—where both forward progression and backward clearance are obstructed—the II-I intermediate is passively trapped and accumulates.
